# Land subsidence contributions to relative sea level rise at tide gauge Galveston Pier 21, Texas

**DOI:** 10.1038/s41598-020-74696-4

**Published:** 2020-10-21

**Authors:** Yi Liu, Jiang Li, John Fasullo, Devin L. Galloway

**Affiliations:** 1 Department of Civil Engineering, Morgan State University, Baltimore, MD 21251 USA; 2 Climate and Global Dynamics Lab, National Center for Atmospheric Research, Boulder, CO 80305 USA; 3 Water Mission Area, Earth System Processes Division, U.S. Geological Survey, Indianapolis, IN 46278 USA

**Keywords:** Climate sciences, Hydrology, Natural hazards, Ocean sciences, Solid Earth sciences

## Abstract

Relative sea level rise at tide gauge Galveston Pier 21, Texas, is the combination of absolute sea level rise and land subsidence. We estimate subsidence rates of 3.53 mm/a during 1909–1937, 6.08 mm/a during 1937–1983, and 3.51 mm/a since 1983. Subsidence attributed to aquifer-system compaction accompanying groundwater extraction contributed as much as 85% of the 0.7 m relative sea level rise since 1909, and an additional 1.9 m is projected by 2100, with contributions from land subsidence declining from 30 to 10% over the projection interval. We estimate a uniform absolute sea level rise rate of 1.10 mm ± 0.19/a in the Gulf of Mexico during 1909–1992 and its acceleration of 0.270 mm/a^2^ at Galveston Pier 21 since 1992. This acceleration is 87% of the value for the highest scenario of global mean sea level rise. Results indicate that evaluating this extreme scenario would be valid for resource-management and flood-hazard-mitigation strategies for coastal communities in the Gulf of Mexico, especially those affected by subsidence.

## Introduction

**Figure 1:**
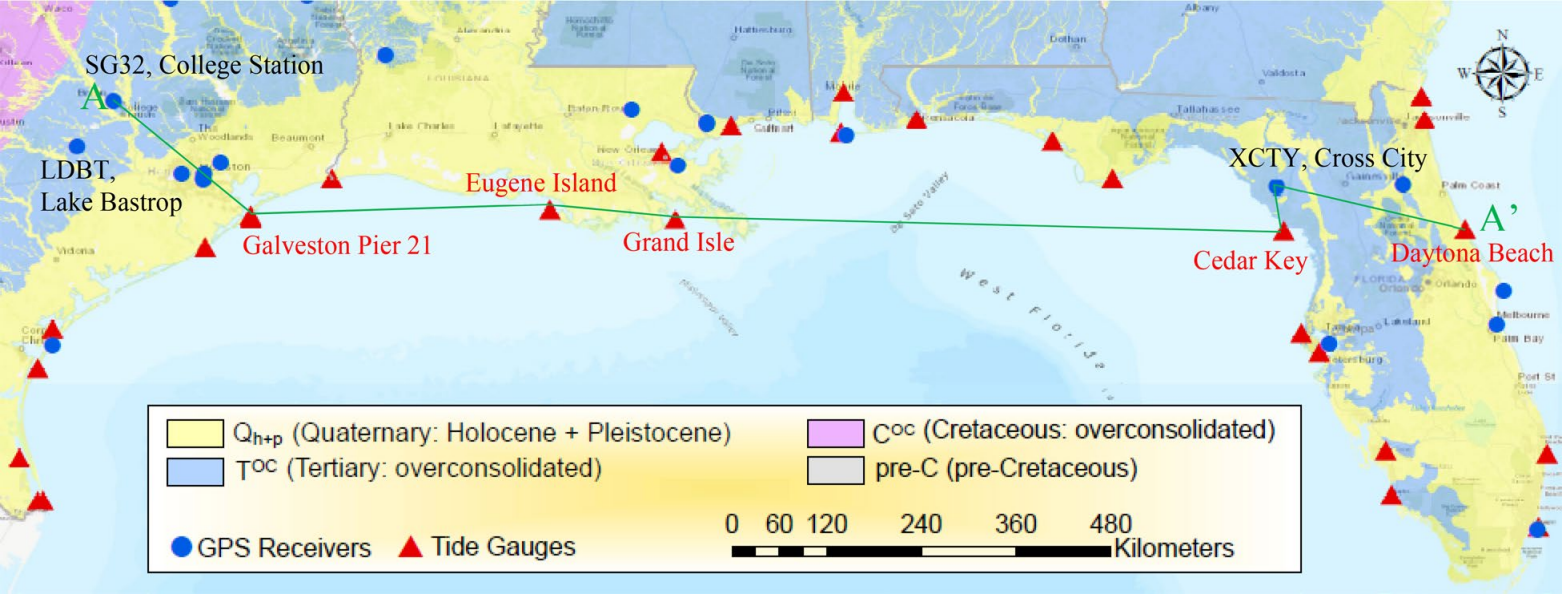
Geological map with tide gauges and GPS stations along the Gulf of Mexico in the United States of America (Based on U.S. Geological Survey digital geological map^10^ with the GIS data from NOAA^4^, JPL (Jet Propulsion Laboratory)^11^ and GLOSS^12^). Geological cross-section A-A′ is shown in Fig. 2. (mapped by using ArcGIS 10.6.1; https://desktop.arcgis.com/en/).

Many severe hurricane-induced urban floods have occurred in U.S. coastal communities along the Gulf of Mexico in recent decades (including Harvey (2017), Isaac (2012), Ike (2008), Gustav (2008), Katrina (2005), Rita (2005), and Ivan (2004)). The Great Galveston hurricane on Sept. 8, 1900, killed more than 6,000 people and destroyed approximately 3,000 homes at Galveston City with a 4.6 m storm surge that swept through the city^1^. The latest severe flood caused by Hurricane Harvey (2017) in the Houston–Galveston region was regarded as one of the costliest disasters in U.S. history, with damages exceeding $100 billion. Flood risk in this region is elevated in part because relative sea level rise (RSLR^2^) in the Galveston Bay is about four times greater than global mean sea level rise (GMSLR^2^). RSLR, measured at any tide gauge, is the combination of absolute sea level rise (ASLR) due to global warming and land subsidence (LS) due to tectonic downward movement, subsurface fluid withdrawal and creep of soil and rock. Lying within one of the globe’s key hot spots of sea level rise^3^, tide gauge Galveston Pier 21 is one of 22 tide gauges along the U.S. coast of the Gulf of Mexico in the U.S. (these gauges and five others along the Atlantic coast of Florida are shown in Fig. 1). This gauge has the longest tide record (110 years, 1909 − 2018 are analyzed in this study) since 1904 among the 27 gauges. Linear trends of relative sea level over time for the entire period of record for each gauge vary from 2.13 mm/a at Cedar Key, Florida to 9.65 mm/a at Eugene Island, Louisiana^4^, where ‘a’ in the denominator denotes annum throughout the paper. Sea level in Galveston Bay has risen about 71 cm with a linear trend of 6.51 mm/a at tide Gauge Galveston Pier 21 since 1904^4^. This RSLR rate is 3.8 times larger than the GMSLR rate of 1.7 mm/a^5^. An additional 0.3–1.2 m of GMSLR is projected to occur by 2100^5^. While current and future GMSLR is associated with global warming^5^, the primary cause of local RSLR in the Houston–Galveston region during the past 50 years has been LS associated with groundwater extraction. In the next several decades, storm surges and high tides are likely to combine with GMSLR and LS to further increase flooding in many regions^6,7^. GMSLR will continue in response to the current state of global warming beyond 2100 because the oceans take a very long time to equilibrate with warmer conditions at the Earth’s surface^6^. Ocean waters will therefore continue to warm and sea levels will keep rising for many centuries^8^. Recent research indicates that present day carbon dioxide levels are sufficient to cause Greenland to melt completely over the next several thousand years^9^. Therefore, the Houston–Galveston region will likely continue to be one of the world’s large coastal communities that is most susceptible to coastal and inland flooding from hurricanes and other extreme storms. Improved understanding of RSLR, particularly contributions from LS, is fundamental in adapting resource-management and flood-mitigation policies.

**Figure 2:**
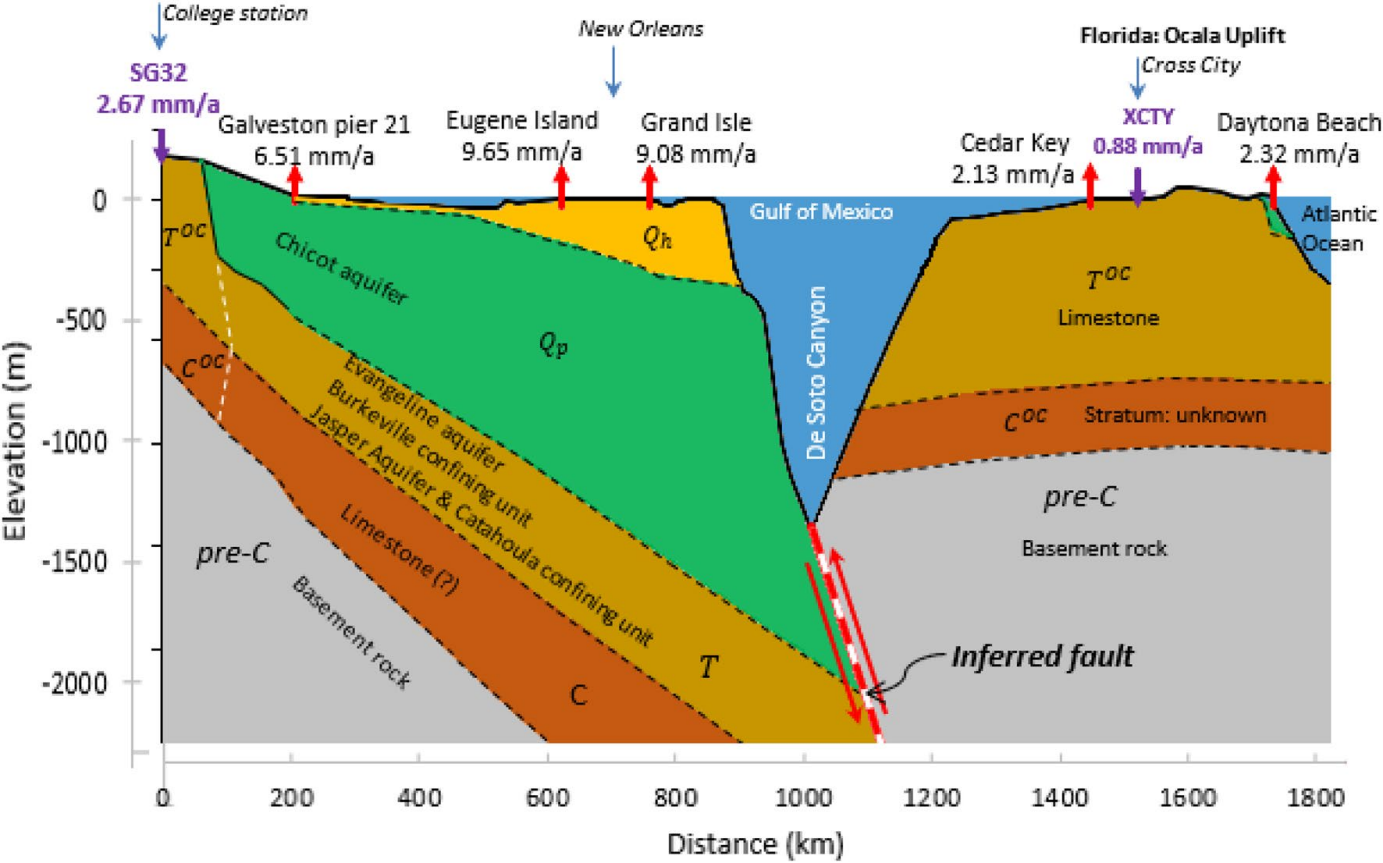
Geological cross-section showing locations of GPS receivers, and tide gauges from College Station to Galveston Pier 21, TX to Eugene Island and Grand Isle, LA to Cedar Key, Cross City and Daytona Beach, FL (A-A′ in Fig. 1). GPS receivers SG32 and XCTY measure LS_BR_^19^; tide gauges Galveston Pier 21, Eugene Island, Grand Isle, Cedar Key and Daytona Beach measure RSLR. The dashed lines are based on previous studies^32–34^. Land-surface elevation is from Google Earth Pro. RSLR values are from NOAA^4^. GPS LS values are from GLOSS^12^ and JPL^11^. Upward pointing red arrows indicate RSLR at tide gauge stations and purple downward pointing arrows indicate LS at GPS stations. See Table 1 for geological layer symbols and Supplementary Fig. S2 for aquifer systems in the Houston–Galveston region.

The ASLR^7,13,14^ is equivalent to eustatic sea level rise (SLR)^15,16^ or global-mean geocentric SLR^2^ and attributed to global warming. In this paper it is assumed that LS^7,13,14,17^ includes two components LS_BR_ and LS_nBR_, where LS_BR_ is subsidence contributed from bedrock systems or non-compacting strata owing to tectonic subsidence (TS)^18–20^ and creep of bedrock systems (SC_BR_)^21^; and LS_nBR_ is subsidence contributed from the compaction of susceptible (compressible, non-bedrock) aquifer systems owing to primary compaction (SPC) caused by subsurface fluid withdrawal^22–27^ and creep of these aquifer systems (SC_nBR_)^28–30^. Thus, RSLR = ASLR + LS, where LS = LS_BR_ + LS_nBR_; LS_BR_ = TS + SC_BR_; and LS_nBR_ = SPC + SC_nBR_ (see Table 1). By application, due to geological similarity (see Fig. 2), LS = LS_BR_ + LS_nBR_ = LS_BR_ can be found at the two adjacent locations of tide gauge Cedar Key and GPS station XCTY at Cross City, Florida as LS_nBR_ = 0. Therefore, RSLR = ASLR + LS_BR_ at tide gauge Cedar Key and similarly, RSLR = ASLR + LS_BR_ + (SPC + SC_nBR_) at tide gauge Galveston Pier 21 where LS_BR_ can be measured at GPS station SG32 at College Station, TX.

**Table 1:** Geological type and symbol of tide gauge and GPS receiver site in the study area.

Geological materials		Symbol of station type	Geological time			Stress history		Consolidation degree^d^	Subsidence type	
			Period	Epoch	Start, MYBP	Experienced effective stress (${\upsigma }_{{\text{c}}}^{{\prime}} )$	Current effective stress (${\upsigma }_{{\text{o}}}^{{\prime}} )$			
Geological system	Aquifer systems	${\text{Q}}_{{\text{h}}}$	Quaternary	Holocene	0.00117	Very low	${\upsigma }_{{\text{o}}}^{{\prime}} \approx {\upsigma }_{{\text{c}}}^{{\prime}}$	1	SPC, SC_nBR_	LS_nBR_
		${\text{Q}}_{{\text{p}}}$		Pleistocene	2.58	Low		2		
		${\text{T}}$	Tertiary	Pliocene to Paleocene	66	High		3		
		${\text{C}}$	Cretaceous		145	Very high		4		
		T^OC^	Tertiary	Pliocene to Paleocene	66	High	${\upsigma }_{{\text{o}}}^{{\prime}} < {\upsigma }_{{\text{c}}}^{{\prime}}$	5	SC_nBR_^a^	
		C^OC^	Cretaceous		145	Very high				
	Bedrock system	pre-C	Jurassic to Precambrian		4600	Highest	$\upsigma _{{\text{o}}}^{{\prime }} <\upsigma _{{\text{c}}}^{{\prime }} { }$ ^b^ $\upsigma _{{\text{o}}}^{{\prime }} \approx\upsigma _{{\text{c}}}^{{\prime }}$ ^c^	6	TS, SC_BR_	LS_BR_

LS_nBR_—Land subsidence due to compaction of non-bedrock systems (aquifer systems); LS_BR_—Land subsidence due to TS and SC_BR_ of the bedrock system. SC_nBR_—Subsidence due to creep of non-bedrock systems (aquifer systems); SPC—Subsidence due to primary compaction; TS—Tectonic subsidence; and SC_BR_—Subsidence due to creep of the bedrock system.

^a^SC_nBR_ from the T^OC^ and C^OC^ strata in the human observation period is assumed to be insignificant after a long-term accumulative creep such as the length CD in Supplementary Fig. S3 after an overburden removal event.

^b^In the College Station, Texas area and the region from De Soto Canyon to Daytona Beach, Florida in Fig. 2.

^c^In the region from the west of Galveston Pier 21, Texas to De Soto Canyon, Florida in Fig. 2.

^d^The consolidation degree of geological strata is based on the strata’s stress history in column stress history of this Table 1; 1—Very unconsolidated; 2—Unconsolidated; 3—Semi-consolidated^35^; 4—Highly semi-consolidated; 5—Over semi-consolidated; and 6—Consolidated (Bedrock).

This paper quantifies RSLR at tide gauge Galveston Pier 21 as the combination of ASLR, LS_BR_ and LS_nBR_ based on an estimate of ASLR for the Gulf of Mexico and an analysis of the historical subsidence in the Houston–Galveston region, and forecasts the RSLR in 2100 at tide gauge Galveston Pier 21. Firstly, an estimate of ASLR for the Gulf of Mexico is computed based on the estimated LS at tide gauge Cedar Key, Florida, where it is assumed that only LS_BR_ contributes to LS because LS_nBR_ is considered negligible owing to a lack of groundwater-level declines and an overconsolidated stress condition^31^. Next, the estimated ASLR is used along with estimates of each of the two components of LS (LS_BR_ and LS_nBR_) to evaluate RSLR in the historical record at tide gauge Galveston Pier 21 and project RSLR to 2100. SPC and SC_nBC_ resulting from compressible aquifer systems at tide gauge Galveston Pier 21 are estimated through analysis of (1) historical LS_nBR_ measurements and coupled groundwater-flow and LS simulation results; (2) annual mean RSLR data from long-term tide gauge records (Supplementary Fig. S1A); (3) the uniform ASLR in the Gulf of Mexico; and (4) LS_BR_ in the Houston–Galveston region estimated from the measurements at GPS station SG32 (Fig. 1).

## Results

### Absolute sea level rise (ASLR) in Gulf of Mexico

Of the 22 tide gauges shown in Fig. 1 along the Gulf coast of the U.S., 21 are situated on more compressible Quaternary strata. Only tide gauge Cedar Key (Figs. 1 and 2) and its nearby reference benchmarks are situated directly over outcropped over semi-consolidated (its geohistorical overburden pressure is larger than the current overburden pressure; see Table 1) Tertiary limestone (T^OC^), for which SPC and LS_nBR_ are negligible due to no significant decline of groundwater level and the removal of geohistorical overburden layers, i.e., the absence of Quaternary strata Q_h_ and Q_p_ in Fig. 2. Moreover, the creep magnitude during the period of human observation is negligibly small (e.g., the length C-D in Supplementary Fig. S3). Only one GPS station XCTY is established on the same limestone, 55 km distant from the Cedar Key gauge. LS (≈LS_BR_) measured at XCTY (Supplementary Fig. S4A) and the minimum RSLR gauged at Cedar Key (Supplementary Fig. S1B) are used to estimate a uniform ASLR in the Gulf of Mexico before 1992.

**Figure 3:**
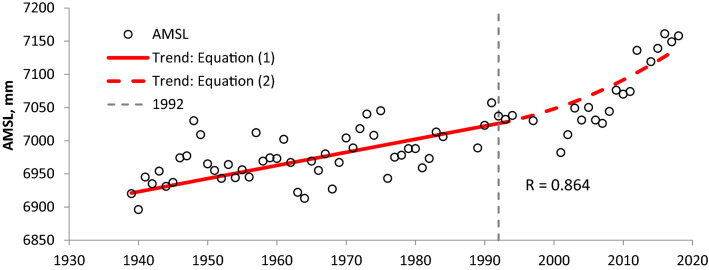
Annual mean sea level (AMSL) and simulated trends (Eqs. (1) and (2)) at tide gauge Cedar Key, Florida (Data source: PSMSL^38^ based on NOAA tide records). (Note: Datum is RLR (revised local reference) defined to be approximately 7000 mm below mean sea level by PSMSL at each tide gauge).

Assuming tide gauge Cedar Key measures RSLR comprising ASLR and LS (where LS = LS_BR_ owing to negligible LS_nBR_), and that the LS measured at the GPS station XCTY at Cross City, FL (where LS≈LS_BR_) can be applied at tide gauge Cedar Key, then ASLR can be evaluated by subtracting the LS measurement at XCTY from RSLR measured at Cedar Key. Height time series at GPS station XCTY during 2004–2013 is shown in Supplementary Fig. S4A. A long-term LS rate of 0.88 mm/a was derived by SONEL^12^ (Système d'Observation du Niveau des Eaux Littorales) at GPS station XCTY using Ellipsoid GRS80^36^. Piecewise trend equations (1) and (2) applied to simulate annual mean sea level at tide gauge Cedar Key with a regression coefficient (R) of 0.864 (Fig. 3) follow through PEST^37^ mimic linking a Fortran code of equations (1) and (2) for identifying best parameter values:

1 $${\text{AMSL}} = 1.98{\text{t}} + 3081.85,\;{\text{ t}} \in [1939\;{\text{to}}\;1992]$$

2 $${\text{AMSL}} = 0.09276\left( {{\text{t}} - 1992} \right)^{2} + 1.98{\text{t}} + 3081.85,\;{\text{ t}} \in (1992\;{\text{to}}\;2018]$$

where AMSL is annual mean sea level (PSMSL^38^) in mm and t is time in years. The constant (linear) RSLR rate at tide gauge Cedar Key is 1.98 mm/a from equations (1) and (2). A constant (linear) ASLR rate of 1.10 mm/a at tide gauge Cedar Key before 1992, which is selected based on the linear trend of GMSLR before 1992 in the twentieth century and the quadratic trend since 1992^39^, is derived from the difference between the RSLR rate of 1.98 mm/a and the LS rate of 0.88 mm/a. This value is used as the regional constant (linear) ASLR rate for the Gulf of Mexico and is represented by ${\text{a}}_{\text{r}}$ in supplementary equation set (S1) for tide gauge Galveston Pier 21. An ASLR acceleration at tide gauge Cedar Key after 1992 of 0.1856 mm/a^2^ (2 × 0.09276) is estimated from Eq. (2).

### Land subsidence due to tectonics (LS_BR_) in the Houston–Galveston region

The following strata underlie tide gauge Galveston Pier 21 (Fig. 2, Supplementary Fig. S6; Table 1) and its nearby reference benchmarks (GPS station TXGA and extensometer site Texas City-Moses Lake; see Supplementary Fig. S6): 1. Quaternary unconsolidated layer which includes surficial thin Holocene sediments and the more than 400-m thick Pleistocene deposits constituting the Chicot aquifer; 2. Tertiary semi-consolidated deposits which includes the Evangeline aquifer, Burkeville confining unit, Jasper aquifer and the Catahoula confining system with a total thickness of as much as 350 m; 3. Cretaceous highly-semi-consolidated limestone^10^; and 4. pre-Cretaceous rocks referred to here as basement rock. The Tertiary strata are outcropped and (or) uplifted in the western area such as at College Station, Texas (Fig. 2 and Supplementary Fig. S2). The outcropped and (or) uplifted Tertiary strata are over semi-consolidated (Figs. 1 and 2 and Supplementary Fig. S2). Therefore, LS at Galveston Pier 21 should include LS_BR_ from pre-Cretaceous and its underlying strata (bedrock systems) and LS_nBR_ from both SPC of the compressible Chicot and Evangeline aquifer systems and the Burkeville confining unit due to groundwater withdrawal, and SC_nBR_ of all the compressible Quaternary, Tertiary and Cretaceous strata.

GPS station SG32, established on the outcropped over semi-consolidated Tertiary Yegua formation (Ey) comprising sandstone, clay and lignite deposits with a thickness of 229–305 m, measured land elevation changes from 2003 to 2014. Supplementary Fig. S8 shows the silty sandstone outcrops in College Station, Texas. The uplifted Cretaceous layer and pre-Cretaceous basement rocks underlie the Tertiary strata. JPL’s height time series at GPS station SG32 from 2003 to 2014 is shown in Supplementary Fig. S4C from which a long-term constant (linear) LS rate of 2.67 mm/a^11^ is derived. GPS station LDBT (Fig. 1), 107 km southwest of GPS station SG32, is anchored on the outcropped, over semi-consolidated Tertiary Calvert Bluff formation (Ecb) composed of mudstone. SPC is negligible in the Ecb because no fluids are available for development from the formation. A long-term constant (linear) LS rate of 2.68 mm/a was derived^11^ at this station using GPS station LDBT elevation data from 2003 to 2009 (Supplementary Fig. S4D). LDBT is 257 km from Galveston Pier 21. A regional LS_BR_ value can be evaluated from the measured LS (height changes) on over semi-consolidated strata underlying GPS station SG32 in Fig. 2 and Supplementary Figs. S3 and S4 due to negligible primary compaction (SPC≈0) and creep (SC_nBR_≈0) in these materials and the absence of geohistorical overburden layers – Quaternary strata (Q_h_ and Q_p_ in Fig. 2). Moreover, the creep magnitude of these strata (SC_nBR_) during the human observation period under the overconsolidation stress condition at GPS station SG32 is negligible (see the path C-D in Supplementary Fig. S3). The similar LS values measured at the two GPS stations support the estimate of LS_BR_ in the Houston–Galveston region. LS_BR_ at tide gauge Galveston Pier 21 has a value of 2.67 mm/a used as coefficient ${\text{s}}_{\text{BR}}$ in supplementary equation set (S1) to compute the RSLR at this tide gauge underlain by compressible aquifer systems.

### Subsidence due to primary compaction (SPC) at tide gauge Galveston Pier 21

During 1918 and subsequent years, millions of barrels of oil were removed from the Goose Creek Oilfield (see location in Supplementary Fig. S6), 46 km northwest of Galveston Pier 21^25^. Between 1918 and 1926 a maximum LS of 115 mm/a (92 cm for 8 years) was measured in the oilfield. No subsidence attributed to production in the oilfield was observed within 40 km of Galveston Pier 21. This implies that SPC attributed to oil and gas production from the oilfield was negligible at Galveston Pier 21. By 1937, groundwater levels were falling in a growing set of gradually coalescing cones of depression centered on the areas of intensive groundwater withdrawal from the Chicot and Evangeline aquifers^25^. One of these areas was in Texas City about 16 km from Galveston Pier 21. About 1.6 mm/a LS (6 cm for 37 years) was estimated at Galveston Pier 21 from regional leveling measurements during 1906 to 1943 (Supplementary Fig. S5A). An average LS of 0.85 mm/a (cumulative LS of 4 cm for 47 years) occurring at Texas City from 1890 to 1937 was simulated using a coupled groundwater-flow and subsidence model HAGM.2013 (Supplementary Fig. S5D)^32^. This indicates that prior to about 1937 SPC at Galveston Pier 21 was small (probably much less than 0.85 mm/a) because this location is on the periphery of the subsidence depression in Texas City. About 7.0 mm/a LS rate (21 cm of SPC for 30 years) at Galveston Pier 21 was estimated during 1943–1973 (Supplementary Fig. S5B). Less than 6.8 mm/a SPC (15 cm of SPC for 22 years) was estimated during 1973–1995 (Supplementary Fig. S5C). Though no significant subsidence at Texas City was simulated by HAGM.2013^32^ during 1974–2009 (Supplementary Fig. S5D), 7.5 mm/a LS (6 cm of subsidence (compaction) for 8 years) was observed during 1973–1981 from the Texas City–Moses Lake borehole extensometer (see location in Supplementary Fig. S6, Supplementary Fig. S5D). No demonstrable subsidence was observed after 1983 at Texas City. Therefore, it is reasonable to assume that SPC occurred principally during 1937–1983 at Galveston Pier 21. Combining with the analysis of annual mean RSLR at tide gauge Galveston Pier 21 (Fig. 5), it is determined that 1937 and 1983 represent the initial year and the ending year, respectively, of SPC at this tide gauge.

### Subsidence due to creep of non-bedrock aquifer system (SC_nBR_) estimated based on borehole-extensometer data in the Houston–Galveston region

**Figure 4:**
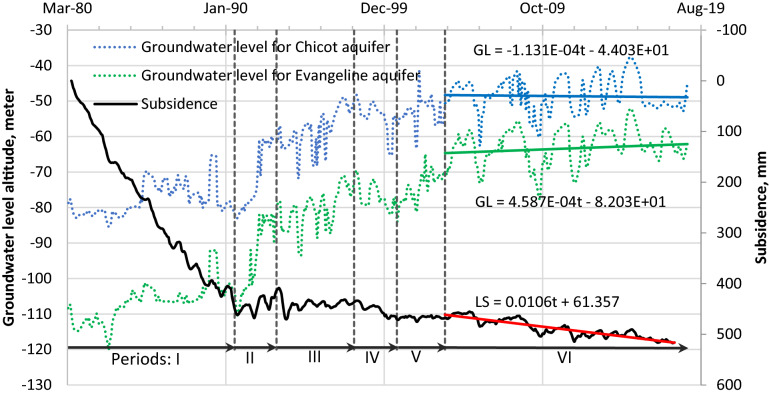
Inelastic SPC ended in about 2000 and SC_nBR_ became apparent at extensometer Southwest in Houston when groundwater-level (GL) trends were stable. I: Inelastic SPC dominated LS; II: elastic rebound dominated LS; III: SC_nBR_ and SPC offset rebound; IV: elastic SPC and SC_nBR_ > 0 while inelastic SPC approached 0; V: elastic rebound offset SC_nBR_; and VI: SC_nBR_ apparent in trend line (red line) while SPC absent. Slope of SC_nBR_ trend (red line) is 0.0106 mm/d or 3.87 mm/a. (modified from^30^).

A previously established instrumentation system monitoring LS_nBR_ of compressible aquifer systems in the Houston–Galveston region includes 11 borehole extensometer stations comprising 13 borehole extensometers^32^ (Supplementary Fig. S6). Two of the stations Baytown and Clear Lake have shallow and deep borehole extensometers and each of other 9 stations have only one borehole extensometer. Supplementary Fig. S7 shows measured LS in terms of compaction measured at each extensometer from the 1970s or 1980s to 2017. Supplementary Fig. S7 shows the negligibly variable SC_nBR_ of Quaternary and Tertiary strata (Q_p_ + T) after inelastic SPC ended around 2000, for various periods (Supplementary Table S1) from the mid-to late-2000s onward during which groundwater levels in the Chicot and Evangeline aquifers were stable (Supplementary Table S1) owing to effective groundwater resource management practices^30^. SC_nBR_ rates range from 0.08 to 8.49 mm/a (Supplementary Table S1) (corresponding to the slopes of the SC_nBR_ trendlines which range from 2.22 × 10^–4^ to 2.327 × 10^–2^ mm/d in Supplementary Fig. S7)^30^, where ‘d’ in the denominator denotes day throughout the paper. Determination of 3.87 mm/a SC_nBR_ at extensometer Southwest is shown in Fig. 4. These results indicate that the SC_nBR_ from the Quaternary unconsolidated and Tertiary semi-consolidated strata also occurs at the location of tide gauge Galveston Pier 21. Note, the SC_nBR_ of Holocene strata (Q_h_) in the Mississippi Delta was found to be as much as 5 mm/a based on analysis of a series of radiocarbon-dated sediment cores^29^.

### Subsidence due to primary compaction (SPC) and absolute sea level rise (ASLR) acceleration estimated from tide gauge records at Galveston Pier 21

**Figure 5:**
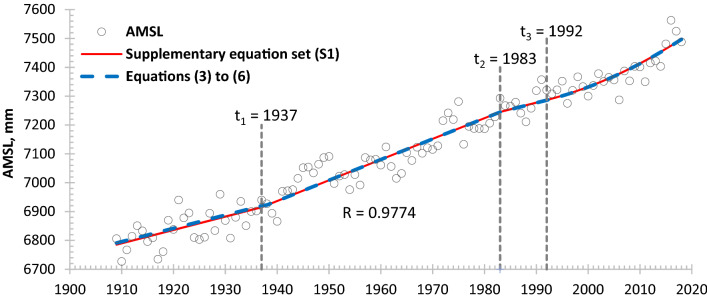
Annual mean sea level (AMSL) and simulated trend lines at tide gauge Galveston Pier 21 (Data source: PSMSL^38^ based on NOAA tide gauge records).

ASLR of 1.10 mm/a (${\text{a}}_{\text{r}}$ in supplementary equation set (S1)) and LS_BR_ of 2.67 mm/a (${\text{s}}_{\text{BR}}$ in supplementary equation set (S1)) contributed to RSLR at Galveston Pier 21 for the period of record analyzed as noted above. Also noted above, SPC occurred during 1937–1983 at the location of tide gauge Galveston Pier 21, and its value was determined by differencing the linear trend of RSLR during 1937–1983 from the linear trend during 1909–1937 and 1983–1992 when SPC was considered negligible at the location of the tide gauge. A piecewise trend of the AMSL, expressed by equations (3) to (6) was obtained using PEST^40^ simulation to estimate all other coefficients (i.e., −1994.78 mm, 7.16 mm/a and −6953.15 mm, −1877.03 mm, and 0.1349 mm/a^2^ applied in equations (3) to (6)) with a regression coefficient of 0.98 (blue dashed line, Fig. 5) as below:

3 $${\text{AMSL}} = 4.60{\text{t}} - 1994.78,\;{\text{t}} \in [1909\;{\text{to}}\;1937]$$

4 $${\text{AMSL}} = 7.16{\text{t}} - 6953.15,\;{\text{t}} \in (1937\;{\text{to}}\;1983]$$

5 $${\text{AMSL}} = 4.60{\text{t}} - 1877.03,\;{\text{t}} \in (1983\;{\text{to}}\;1992]$$

6 $${\text{AMSL}} = 4.60{\text{t}} - 1877.03 + 0.1349\left( {t - 1992} \right)^{2} ,\;{\text{t}} \in (1992\;{\text{to}}\;2018]$$

Equation (4) shows a constant (linear) RSLR rate of 7.16 mm/a during 1937–1983, which is increased by groundwater withdrawal, from 4.60 mm/a before 1937 and after 1983. The difference of 2.56 mm/a is the estimated SPC rate at Galveston Pier 21 during 1937–1983 with a cumulative SPC of 118 mm for the subperiod. Equation (6) is quadratic with an ASLR acceleration of 0.270 mm/a^2^ (2 × 0.1349) in addition to a constant (linear) rate of 4.60 mm/a, which is the combination of ASLR, LS_BR_ and the SC_nBR_ rates determined for the period 1992–2018. Note: The negligibly-variable SC_nBR_ is assumed in equations (3) to (6) for identification of SPC (see details in section of SC_nBR_ in Supplementary Information). This acceleration is considered to be driven by climate change^41^.

### Variable subsidence due to creep of non-bedrock aquifer systems (SC_nBR_) estimated from tide gauge records at Galveston Pier 21

For a more accurate projection of long-term RSLR, variable SC_nBR_ is considered in the piecewise supplementary equation set (S1) of RSLR with parameter ${\text{C}}_{\text{H}}$ based on the creep theory of compressible sedimentary materials^31,42^. Two unknown parameters ${\text{C}}_{\text{H}}$ and ${\text{C}}$ in the supplementary equation set (S1) can be evaluated as 3825.51 mm and 6780.61 mm, respectively, using PEST^37^ simulation. The other parameters are given as the following:${\text{a}}_{\text{r}}$ = 1.10 mm/a, ${\text{s}}_{\text{BR}}$ = 2.67 mm/a, ${\text{p}}_{\text{l}}$= 2.56 mm/a ,${\text{a}}_{\text{c}}$ = 0.270 mm/a^2^, ${\text{t}}_{0}$ = 1908, ${\text{t}}_{1}$ = 1937, ${\text{t}}_{2}$ = 1983, and ${\text{t}}_{3}$ = 1992, where:${\text{a}}_{\text{r}}$ denotes a reginal uniform ASLR rate; ${\text{s}}_{\text{BR}}$ and ${\text{p}}_{\text{l}}$, the annual rates of LS_BR_ and SPC, respectively; a_c_, the regional ASLR acceleration rate; and ${\text{t}}_{0}$, ${\text{t}}_{1}$, ${\text{t}}_{2}$ and ${\text{t}}_{3}$, the start times of each of the four subperiods. The red solid line in Fig. 5 shows the trend line of RSLR found with supplementary equation set (S1). From 1983 to 1992 estimated SC_nBR_ rates range narrowly from 0.84 to 0.83 mm/a, which is comparable to 0.83 mm/a derived from Eq. (5) for example, by removing the ASLR of 1.10 mm/a and LS_BR_ of 2.67 mm/a from the computed RSLR rate of 4.60 mm/a. The estimated variable SC_nBR_ rates computed using the formula $\text{0.4343}{\text{C}}_{\text{H}}\text{/t}$ were 0.87 mm/a in 1909, 0.82 mm/a in 2018 and 0.79 mm/a in 2100.

### Projection of mean relative sea level rise (RSLR) at tide gauge Galveston Pier 21

Figure 5 shows that sea level has risen by about 0.7 m since 1909 at Galveston Pier 21. Supplementary equation set (S1) with all parameter values identified above was used to project RSLR at tide gauge Galveston pier 21 from 2018 to 2100. The projected RSLR of 1.9 m is about 90% and 146% of the highest (2.1 m) and intermediate-high (1.3 m) scenarios of GMSLR^39^, respectively. The projected ASLR acceleration of 0.270 mm/a^2^ is about 87% and 155% of the highest (0.312 mm/a^2^) and intermediate-high (0.1742 mm/a^2^) GMSLR scenarios, respectively. The projected ASLR acceleration of 0.1856 mm/a^2^ computed previously at tide gauge Cedar Key for the same period is about 60% and 107% of the highest and intermediate-high scenarios of GMSLR^39^, respectively. Therefore, the results in this paper indicate that it may be prudent to consider the highest scenario of GMSLR in resource-management and flood-hazard-mitigation strategies for coastal communities in the Gulf of Mexico, especially those affected by LS.

### Contributions to relative sea level rise (RSLR) at tide gauge Galveston Pier 21

**Figure 6:**
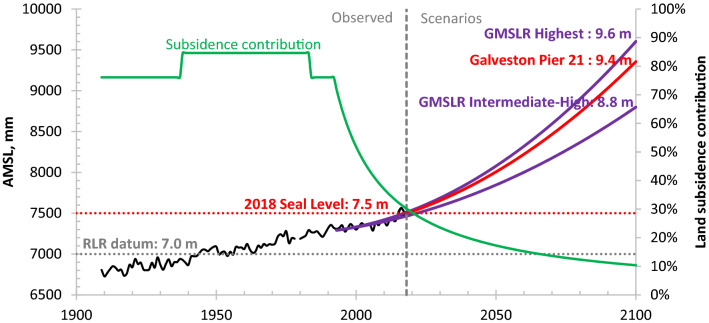
Projected annual mean sea level (AMSL) and estimated land subsidence (LS) contribution to relative sea level rise (RSLR; RLR: See note in Fig. 3 caption) at tide gauge Galveston Pier 21 shown with projections for the highest and intermediate-high scenarios of global mean sea level rise (GMSLR)^39^.

RSLR was computed for tide gauge Galveston Pier 21 for four subperiods during the period 1909–2100 using supplementary equation set (S1) (note: the first three subperiods are the same as used in equations (3) to (6); the fourth subperiod is different from, but inclusive of, the fourth subperiod [1992–2018] used in equations (3) to (6)). Contributions from ASLR, LS_BR_, SPC and SC_nBR_ to RSLR vary in different subperiods and are estimated to be 24, 58, 0 and 18% of the 4.63 mm/a RSLR during 1909–1937; 15, 37, 36 and 12% of the 7.18 mm/a RSLR during 1937–1983; and 24, 58, 0 and 18% of the 4.61 mm/a RSLR during 1983–1992, respectively (see Table 2). Thus, ASLR contributed an estimated 15–24% to RSLR at Galveston Pier 21 from 1909 to 1992, while LS contributed 76–85%. The estimated LS contribution (Fig. 6) to RSLR during 1992–2100 decreased from 76% in 1992 to 30% in 2018 and is projected to decrease to 10% by 2100. The estimated LS contribution to RSLR at Galveston Pier 21 in 2000 was 52%. The estimates indicate that LS dominated RSLR in the twentieth century but since 2001, ASLR driven by global warming has dominated RSLR at tide gauge Galveston Pier 21.

**Table 2:** Contributions of ASLR, LS_BR_, SPC and SC_nBR_ to RSLR from 1909 to 1992.

Item	Contribution					
	1909–1937		1937–1983		1983–1992	
	Rate (mm/a)	%	Rate (mm/a)	%	Rate (mm/a)	%
ASLR	1.10	24	1.10	15	1.10	24
LS_BR_	2.67	58	2.67	37	2.67	58
SPC	0.00	0	2.56	36	0.00	0
SC_nBR_	0.86	18	0.85	12	0.84	18
RSLR	4.63	100	7.18	100	4.61	100

## Discussion

Tide gauge Cedar Key is the sole tide gauge station anchored on the over semi-consolidated Tertiary strata with minimal local groundwater development along the U.S. coast of the Gulf of Mexico. Assuming the constant (linear) ASLR rate of 1.10 mm/a with uncertainty of $\pm 0.19$ mm/a (comparable to $\pm 0.18$ mm/a in Supplementary Fig. S1B from NOAA), obtained from tide gauge Cedar Key and the GPS station XCTY, is regionally representative, then the ASLR rate estimated from any other tide gauge along the Gulf coast should be very close to 1.10 mm$\pm 0.19$/a. Tebaldi et al.^7^ assumed ASLR along the U.S. coasts is approximately equal to GMSLR of 1.70 mm/a for estimating LS values at each tide gauge location. To evaluate the representativeness of the ASLR rate estimated for tide gauge Cedar Key, ASLR rate was estimated from tide gauge Galveston Pier 21 and its reference GPS station TXGA, 3 km distant (Supplementary Fig. S6). The LS rate at TXGA of 3.44 mm/a was estimated by SONEL^12^ (see Supplementary Fig. S4B) for the period 2005–2014. This LS is the sum of LS_BR_ and SC_nBR_ at this station because LS_nBR_ represents only SC_nBR_ as there was no SPC. A constant (linear) ASLR rate of 1.16 mm/a was derived by subtracting the LS rate of 3.44 mm/a from the constant (linear) RSLR rate of 4.60 mm/a in Eq. (6), assuming 3.44 mm/a approximately represents the constant (linear) LS rate at Galveston Pier 21 and recognizing that the acceleration of 0.270 mm/a^2^ is only related to global warming. The derived ASLR rate of 1.16 mm/a leads to a difference of 5% relative to 1.10 mm/a estimated at tide gauge Cedar Key. The ASLR rate difference of 0.06 mm/a may be due to differences between the Quaternary and Tertiary strata underlying GPS station TXGA and those underlying tide gauge Galveston Pier 21. However, the similar rates estimated at the two tide gauges support the use of the estimated 1.10 mm/a as a regionally representative rate of ASLR (a_r_ in supplementary equation set (S1)) in the Gulf of Mexico, which is comparable to an ASLR rate of 1.11 mm/a estimated from the tide gauge in Baltimore^43^. (Note: Wang et al.^44^ estimated ASLR for the Gulf of Mexico using a new reference frame but the results presented for ASLR for the same tide gauges analyzed in this article are not comparable to our estimate that is based on tide gauge Cedar Key for the period before 1992, because the measured LS (designated as VLM in their Table 2) in Wang et al.^44^ was relative and not based on an absolute reference frame such as GRS80 from SONEL used in this study).

If only a single linear equation is used to simulate the RSLR trend for the entire period of record (1908–2018), the effects of LS (particularly SPC) and global warming acceleration may not be accounted for in the analysis. Supplementary Figs. S1A and S1B show linear RSLR trends of 6.51 mm/a at tide gauge Galveston Pier 21 and 2.13 mm/a at tide gauge Cedar Key. Supplementary Table S1 shows that the resulting ASLR rates are 3.07 mm/a at tide gauge Galveston Pier 21 and 1.25 mm/a at tide gauge Cedar Key, respectively, computed using LS_BR_ measured at GPS station TXGA and LS (LS_BR_ + SC_nBR_) at GPS station XCTY. The 1.25 mm/a value at tide gauge Cedar Key is closer to the previously estimated, pre-1992 regionally representative rate of ASLR in the Gulf of Mexico (1.10 mm/a). The resulting ASLR rate of 3.07 mm/a at tide gauge Galveston Pier 21 differs by 146% relative to the 1.25 mm/a value at tide gauge Cedar Key. This large difference underscores the importance of accounting not only for the historical (pre-1992) SPC but also the ASLR acceleration since 1992 when estimating the pre-1992 linear trend of ASLR.

Nearly identical LS_BR_ rates of 2.67 and 2.68 mm/a were measured at GPS stations SG32 (Supplementary Fig. S4C) and LDBT (Supplementary Fig. S4D), respectively, due to negligible SPC and SC_nBR_ at these station locations. The two stations are 107 km apart (Fig. 1). From 2005 to 2014 the LS rate at GPS station TXGA is 3.44 mm/a (Supplementary Fig. S4B) where SPC is absent, the SC_nBR_ rate at GPS station TXGA (see location in Supplementary Fig. S6) of 0.77 or 0.76 mm/a was evaluated by subtracting the LS_BR_ of 2.67 mm/a (Supplementary Fig. S4C) or 2.68 mm/a (Supplementary Fig. S4D) at GPS stations SG32 or LDBT from that at GPS station TXGA, assuming GPS station TXGA is located in the same tectonic zone as GPS stations SG32 and LDBT (see locations in Supplementary Fig. S6). Compared to the SC_nBR_ rate of 0.83 mm/a estimated at tide gauge Galveston Pier 21 from 2005 to 2014, a difference of about 0.06 or 0.07 mm/a in the SC_nBR_ rate at GPS station TXGA is reasonable due to geological material variation between tide gauge Galveston Pier 21 and GPS station TXGA, located 3 km apart. The above analysis demonstrates spatial stability of the estimated LS_BR_ in the Houston–Galveston region and the associated insights that can be gained regarding contributions to RSLR in the vulnerable region. The LS rates at the four GPS stations (i.e., XCTY in Florida, and SG32, LDBT and TXGA in Texas) are derived from a short period 2003–2014 but work well systematically with the RSLR trend for the longer period 1909–2018. This indicates that the LS rates in different tectonic zones, from Florida to the Houston–Galveston region, Texas (Fig. 2), may also be temporally stable within short time scales of the observations compared to the geological time scale of tectonics.

The analyses here for the Galveston Pier 21 tide gauge show that for the period record RLSR is dominated by SPC attributed to groundwater-level declines accompanying groundwater extraction. However, the magnitude of historical aquifer-system compaction and land subsidence in the Houston–Galveston region in inland and other coastal locations (Supplementary Figs. S5 and S7) is far greater than that experienced at the location of the Galveston Pier 21 tide gauge and GPS station TXGA. This indicates that potential impacts of subsidence and RSLR in terms of coastal and inland flooding are likely greater in other areas of this region. Further, the variable spatial and temporal distributions of historical subsidence that arise from the variable distributions of compressible sediments, hydraulic properties in the aquifer systems, and groundwater extractions from the aquifer systems, result in variable potential impacts of subsidence in the region. Another related point is that the RSLR projections for tide gauge Galveston Pier 21 (Fig. 6) assume no changes in future management of groundwater resources in the region. Unlike ASLR, RSLR with substantial contributions from land subsidence can vary locally and can change quickly in response local changes in groundwater extraction. These variabilities indicate that in coastal regions where SPC is an important contributor to RSLR, a more complete vulnerability assessment is needed, one that accounts for the historical and future subsidence and potential future groundwater management practices.

## Materials and methods

### Identification of geological and hydrogeological conditions at tide gauges and GPS stations

A tide gauge measures ASLR and LS and a GPS receiver at the tide gauge’s paired reference station measures LS. Where both the tide gauge and paired reference station are seated on basement rocks or on over semi-consolidated sediments without significant SPC and LS_nBR_, the LS at both sites has only the component of LS_BR_. In contrast, where both the tide gauge and its paired reference station are seated on unconsolidated and/or semi-consolidated sediments, LS constitutes LS_nBR_ (SPC and SC_nBR_) and LS_BR_. Geological and hydrogeological data were used to determine the LS components measured at tide gauges and their paired GPS stations (Table 1).

### Identification of regional absolute sea level rise (ASLR) before 1992

In general, ASLR can be determined by RSLR minus LS that are measured with tide gauges and their nearby GPS stations, respectively. Due to complexity of geological and geohydrological conditions and stress history, LS varies at different locations. ASLR was estimated at tide gauges Cedar Key and Galveston Pier 21 using ASLR = RSLR − LS. LS at tide gauges was estimated using LS = LS_nBR_ + LS_BR_, where LS_nBR_ = SPC + SC_nBR_ and LS_BR_ = TS + SC_BR_. TS was estimated from measurements at GPS stations anchored on bedrock (XCTY for gauge Cedar Key; and SG32 and LDBT for gauge Galveston Pier 21), assuming SC_BR_ was negligible over the human time-scale of observations, thus LS at the GPS stations could be represented by LS = LS_BR_ = TS. These values for LS = LS_BR_ were translated to the tide gaging stations and used to compute ASLR at those gaging stations. At tide gauge Cedar Key where LS_nBR_ was assumed to be negligible, ASLR was estimated using RSLR measured at the gaging station minus the translated estimate of LS = LS_BR_. For tide gauge Galveston Pier 21 anchored in non-bedrock material, it was necessary to also estimate LS_nBR_ = SPC + SC_nBR_ at the tide gaging station.

### Identification of subsidence due to primary compaction (SPC) and absolute sea level rise (ASLR) acceleration

SPC in the Houston–Galveston region accrued during a time period when subsurface fluid was developed. The starting and ending years for a period when SPC occurred at a location was determined by analyzing regional LS leveling data and simulation results as well as annual mean RSLR data. RSLR trends during periods when SPC was either active or inactive were simulated using long-term tide gauge records. ASLR acceleration and RSLR trends in active and inactive SPC periods were further estimated with PEST^40^. Then the SPC rate was estimated from the difference of RSLR trends between SPC active and inactive periods.

### Identification of subsidence due to creep of non-bedrock aquifer system (SC_nBR_)

The existence of SC_nBR_ at a tide gauge station was demonstrated by analyzing aquifer-system compaction measurements and groundwater-level observations in the study area. Negligibly variable SC_nBR_ was used to analyze and estimate SPC before simulation of its variation through compaction due to creep in supplementary equation set (S1)^31,42^ after uniform ASLR rate before 1992, ASLR acceleration, LS_BR_ and SPC were determined at tide gauge Galveston Pier 21.

## Supplementary information

Supplementary Information.

## Data Availability

All data needed to evaluate the conclusions in the paper are present in the paper and/or the Supplementary Information. Additional data related to this paper may be requested from the corresponding author. Groundwater level and borehole extensometer raw data used in this paper are available from the U.S. Geological Survey (USGS) (https://txpub.usgs.gov/houston_subsidence/home/ etc.) Sea level data used in this paper are available from the National Oceanic and Atmospheric Administration (NOAA) (https://tidesandcurrents.noaa.gov/sltrends/sltrends.html) and/or the Permanent Service for Mean Sea Level (PSMSL) (https://www.psmsl.org/). GPS height data used in this paper are available from the Jet Propulsion Laboratory (JPL) of NASA (https://sideshow.jpl.nasa.gov/post/series.html) or SONEL (https://www.sonel.org/?lang=en).
