# Supplementary material for: Land subsidence contributions to relative sea level rise at tide gauge Galveston Pier 21, Texas
Source: Sci Rep. 2020 Oct 21;10:17905. doi: 10.1038/s41598-020-74696-4 (PMC7578811; doi:10.1038/s41598-020-74696-4)
Supplement: Supplementary file 1 — Supplementary Information. [file 41598_2020_74696_MOESM1_ESM.pdf]

Supplementary Information

Land subsidence contributions to relative sea level rise at tide gauge Galveston Pier 21, Texas

Yi Liu[1,*], Jiang Li[1], John Fasullo[2], Devin L. Galloway[3]

[1]Morgan State University, Department of Civil Engineering, Baltimore, MD 21251;
[2]National Center for Atmospheric Research, Climate and Global Dynamics Lab, Boulder, CO 80305;
[3]U.S. Geological Survey, Water Mission Area, Earth System Processes Division, Indianapolis, IN 46278
*Correspondence to: yi.liu@morgan.edu

Supplementary Materials and Methods

Geological materials

Compressible aquifer systems at tide gauge Galveston Pier 21: From northwest to southeast the Houston-Galveston region includes Grimes County with the region's highest elevation of about 122 m, and Montgomery, Waller, Harris and Galveston counties with the lowest elevations of about 0 to 15 m near the coast of the Gulf of Mexico (Fig. 2)[1]. The three primary Quaternary and Tertiary aquifers in the Gulf Coast aquifer system in the region are the Chicot, Evangeline, and Jasper (Fig. 2 and Supplementary Fig. S1)[1–4], which comprise laterally discontinuous deposits of gravel, sand, silt, and clay. The youngest and uppermost Quaternary aquifer, the Chicot aquifer, consists of Holocene- and Pleistocene-age sediments; the underlying Tertiary Evangeline aquifer consists of Pliocene- and Miocene-age sediments; and the oldest and most deeply buried Tertiary aquifer, the Jasper aquifer, consists of Miocene-age sediments (Fig. 2 and Supplementary Fig. S1)[5,6]. The lowermost unit of the Gulf Coast Tertiary aquifer system is the Miocene-age Catahoula confining system, which includes the Catahoula Sandstone. The Catahoula confining system comprises sands in the upper section and clay and tuff interbedded with sand in the lower section. Detailed stratigraphic information regarding the underlying Cretaceous and pre-Cretaceous strata is unavailable in this area. The Cretaceous strata are generally considered semi-consolidated. The pre-Cretaceous strata are bedrocks or basement rocks here (Table 1). A maximum subsidence due to primary compaction (SPC) of 3 m was measured in the Houston-Galveston region and attributed to oil, gas and groundwater development. Subsidence due to creep of aquifer systems or non-bedrock systems (SC_{nBR}) measured from 13 borehole extensometers in this region is shown in Supplementary Table S1 and Supplementary Fig. S7. Thus, the compaction subsidence (LS_{nBR}) in the compressible aquifer systems includes SPC and SC_{nBR}. Land subsidence contributed from the bedrock system (LS_{BR}) (Table 1) includes components of tectonic subsidence (TS) and subsidence due to creep (SC_{BR}) in this region. Therefore, the LS records at tide gauge Galveston Pier 21 and its reference GPS station TXGA includes vertical motion attributed to both LS_{nBR} (SPC + SC_{nBR}) and LS_{BR} (TS + SC_{BR})

Ocala limestone at tide gauge Cedar Key and Cross City: The limestones exposed near Ocala, Marion County, in central peninsular Florida are referred to as the Ocala Limestone[7–10]. The Ocala Limestone consists of nearly pure limestones and occasional dolostones. Numerous disappearing streams and springs occur within these areas. The permeable, highly transmissive carbonates of the Ocala Limestone are one of the most permeable rock units of the Floridan Aquifer System (FAS)[11]. Though details of the underlying Cretaceous and pre-Cretaceous strata are not well known[11], it is believed that the Tertiary limestone and Cretaceous strata were uplifted and are currently over semi-consolidated because their geohistorical overburden materials, Quaternary sediments Q_h and Q_p, were removed. Because minimal groundwater development has occurred in the Cedar Key and Cross City areas, which are 55 km apart within the same tectonic zone, the Cedar Key and Cross City areas should experience identical LS_{BR} (TS + SC_{BR}) and negligible LS_{nBR} (SPC + SC_{nBR}). Both tide gauge Cedar Key and its reference GPS station XCTY are important to identify ASLR from the long-term tide-gauge and GPS station records along the coast of the Gulf of Mexico in the U.S. The other tide gauges and their reference GPS stations along the Gulf of Mexico in the U.S. (Fig. 2) are affected to various degrees by LS_{nBR} and LS_{BR}.

Outcropped over semi-consolidated Tertiary strata at GPS station SG32 in College Station, Texas (Yegua Formation): The Yegua Formation (Ey) consists of sandstone, clay, and lignite with a

thickness of 229 to 305 m[12]. The sandstone near SG32 is shown in Supplementary Fig. S8. In East Texas and along the Gulf Coast to the Rio Grande River, the Yegua Formation overlies the Cook Mt. Formation, and is overlain by the Caddell Formation. SC_{nBR} is negligible in the over semi-consolidated strata ($T^{OC} + C^{OC}$). Minimal groundwater has been developed from this minor aquifer in Texas and no significant groundwater-level decline has been measured by the Texas Water Development Board (TWDB)[13]. Thus, SPC in the strata is negligible. Therefore, land subsidence (LS) measured from GPS station SG32 is attributed solely to LS_{BR}.

Outcropped over semi-consolidated Tertiary strata (Calvert Bluff Formation) at GPS station LDBT: The Calvert Bluff Formation underlying GPS station LDBT 107 km southwest of GPS station SG32 is mostly[14]. The sandstone is medium to fine grained, well sorted, crossbedded, and lenticular. This formation is about 305 m thick. Owing to the over semi-consolidated strata (T^{OC} + C^{OC}) SC_{nBR} is negligible. Because no groundwater has been developed from the strata in the region, SPC is negligible. Thus, LS measured from the paired reference GPS station LDBT is attributed to LS_{BR}.

Piecewise equation of sea level rise (SLR) at tide gauge Galveston Pier 21

Future GMSLR has been projected using the quadratic equation $E_{global}(t)=0.0017t+b_g t^2$, where 0.0017 m/a refers to the trend for the GMSLR determined from observations from 1900 to 1992, with an acceleration-related constant b_g [m/a^2] determined to be 0.0, 2.71×10^{-5}, 8.71×10^{-5}, and 1.56×10^{-4} m/a^2 for the lowest, intermediate-low, intermediate-high, and highest GMSLR scenarios, respectively[15,16]. The highest scenario of GMSLR by 2100 is derived from a combination of estimated ocean warming from the IPCC Fourth Assessment Report (AR4), GMSLR projections and a calculation of the maximum possible glacier and ice sheet loss by the end of the century[15]. The intermediate-high scenario is based on an average of the high end of semi-empirical, GMSLR projections[15]. Semi-empirical projections utilize statistical relations between observed global sea level change, including recent ice sheet loss and air temperature. The intermediate-low scenario is based on the upper end of IPCC AR4 GMSLR projections resulting from climate models using the B1 emissions scenario[15]. The lowest scenario is based on a linear extrapolation of the historical SLR rate derived from tide gauge records beginning in 1900 (1.7 mm/a) [15]. Note, $b_g = 0.5a_g$ where a_g denotes GMSLR acceleration [m/a^2] with values of 0.0, 5.42×10^{-5}, $1,74\times10^{-4}$, and 3.12×10^{-4} m/a^2 for the lowest, intermediate-low, intermediate-high, and highest scenarios of GMSLR, respectively. By comparison, SLR acceleration in the Chesapeake Bay, U.S. was estimated to be about 0.05−0.10 mm/a^2 ($5.0\times10^{-5} - 1\times10^{-4}$ m/a^2) from the 1930s to 2011[17]. The historical and estimated future magnitudes of RSLR are two additional factors for scientists, engineers and planners to consider when devising sustainable solutions involving adaptive engineering, such as seawalls[18–20], levee systems[21–23], and pile-elevated building foundations[24] for future coastal settlements in the U.S. A set of piecewise equations representing a time-dependent model of RSLR at a tide gauge was developed as follows:

$$RSLR(t)=(a_r+s_{BR})(t-t_0)+0.4343C_H \ln(t/t_0)+C,\ t\in(t_0,t_1] \quad (S1\text{-}1)$$

$$RSLR(t)=(a_r+s_{BR})(t-t_0)+0.4343C_H \ln(t/t_0)+p_1(t-t_1)+C,\ t\in(t_1,t_2] \quad (S1\text{-}2)$$

$$RSLR(t)=(a_r+s_{BR})(t-t_0)+0.4343C_H \ln(t/t_0)+p_1(t_2-t_1)+C,\ t\in(t_2,t_3] \quad (S1\text{-}3)$$

$$RSLR(t)=(a_r+s_{BR})(t-t_0)+0.4343C_H \ln(t/t_0)+p_1(t_2-t_1)+0.5a_g(t-t_3)^2+C,\ t\in(t_3, 2100] \quad (S1\text{-}4)$$

where a_r and s_{BR} denote a regional uniform ASLR rate [L/T] and LS_{BR} rate [L/T], respectively; t represents any year [T] such as 1909 in the period 1909-2100; t_0 denotes year 1908, the previous

year of the starting year 1909 for the period of record and the first subperiod 1909–37, and t_1, t_2 and t_3 denote the starting years 1937, 1983 and 1992, respectively for the specified subperiods (1937–83, 1983–92 and 1992–2100) (note: the last subperiod differs from the last subperiod used in equation set (2) in the main text); RSLR(t) signifies the mean RSLR in year, in meters [L]; p_l represent rates of SPC; a_g denotes regional RSLR acceleration [L^2/T]; $C_H = C_\alpha H$ [L] where C_α and H represent the compression coefficient of creep [dimensionless] [25] and total thickness [L] of compressible aquifer systems for variable SC_{nBR}, respectively; and C [L] is a constant the offset of the fit to measured or observed sea level. Model parameters C_H and C were estimated using PEST[26] to optimize the fit to observed RSLR. Values of t_1 and t_2 were determined by analyzing RSLR data with information of regional and local land subsidence, subsurface fluid withdrawal, groundwater level and subsurface fluid-flow simulation. The value of 1992 for t_3 is from Parris et al.[15] and represents the start of the period of ASLR acceleration which extends to 2100 in this analysis. Note, after 1983, the term $p_l(t_1\text{-}t_1)$ in equations (S1-3–4) is constant and represents the SPC contribution for subperiods beyond 1983. The supplementary equation set (S1) for a particular tide gauge reflects the fact that RSLR varies along the U.S. coastline[27]. The U.S. Army Corps of Engineers (USACE)[16] has incorporated sea level change into civil works programs using a single equation that is similar to supplementary equation (S1-4) in Regulation No. 1100-2-8162.

Land subsidence from bedrock systems (LS_{BR})

In this study, LS_{BR} is defined as the portion of land subsidence (LS) attributed to TS and SC_{BR} in bedrock systems. LS_{BR} can be measured at a tide gauge's paired reference GPS station that is anchored on bedrock (pre-Cretaceous) or over semi-consolidated Tertiary and Cretaceous strata for which SPC and SC_{nBR} are negligible. The TS is assumed to be caused by comprehensive intraplate or interplate tectonic activities[28–30], which include regional and local faulting, and glacial isostatic adjustment (GIA)[30]. SC_{BR} represents subsidence due to creep of bedrock systems[31,32]. For purposes of this study, the LS_{BR} trend in terms of an annual rate is assumed to be constant for a specific station during the relatively short time period (human time scale) represented by these analyses. Global GPS height data from 2,567 GPS stations on land are available from the Jet Propulsion Laboratory (JPL)[33]. Of those, 1,961 GPS stations are distributed on the North American Plate and JPL provides trends (rates) computed for each GPS station. The GPS data are also available from SONEL[34]. For example, GPS station SG32 at College Station, Texas, about 206 km northwest of tide gauge Galveston Pier 21 (Figs. 1 and 2), is a reference GPS station used in this study, where LS is attributed solely to LS_{BR}. The trend of height changes over time at SG32 during 2003–14 is -2.67±0.67 mm/a (Supplementary Fig. S4C) representing a rate of LS equal to LS_{BR} of 2.67 mm/a. This value was used to represent LS_{BR} at reference GPS station TXGA for tide gauge Galveston Pier 21 as described in the main article.

Regional ASLR estimation

For purposes of this study, the tectonic conditions in a coastal region were assumed to be identical in a small zone with similar strata. For tide gauges and their paired reference GPS stations where SPC and SC_{nBR} are negligible, LS_{BR} was used to estimate ASLR in a coastal region because those tide gauges were assumed to measure height changes attributed solely to ASLR and LS_{BR}. For example, tide gauge Cedar Key and its reference GPS station XCTY (Figs. 1 & 2) 55 km distant are established over the same outcropped over semi-consolidated (see Table 1) Tertiary limestone (T^{OC})[7], where superscript OC denotes an overconsolidated stress status ($\sigma'_0<\sigma'_c$), where σ'_0 and σ'_c and denote current and historically maximum stresses, for example in Fig. S3)[25]. The NOAA

measured trend in relative sea level referred to as RSLR in this paper is 2.13 mm/a (Fig. Supplementary S1B)[35], while the NOAA measured LS in trend (LS_{BR}) is 0.88 mm/a (± 0.43 mm/a standard deviation) (Supplementary Fig. S4A)[34]. At this gauge, RSLR comprises only ASLR and LS_{BR}, as LS_{nBR} (SPC and SC_{nBR}) in the T^{OC} and the Cretaceous strata C^{OC} (Fig. 2 and Table 1) are negligible. Groundwater development from the unconfined and semiconfined Floridan aquifer along the Gulf coast of peninsular Florida north of Tampa Bay is minimal at Cedar Key in Levy County and Cross City in Dixie County[36,37]. The water table at Cedar Key is hydraulically connected with sea water[36]. Water table measured in USGS Rosewood Tower Well with a depth of 134.7 m, completed in the Tertiary Floridan limestone aquifer 14 km northeast of Cedar Key, ranged from 2.7 to 3.7 m above NGVD29 without significant decline during the available period of record (1976–2011). Six USGS groundwater wells are completed in the Tertiary Floridan aquifer with well depths of 3.8 to 121.3 m in Dixie County where GPS station XCTY is located. No significant trends in groundwater-level decline were evident in the available water-level records (1961–94). Therefore, SPC in the Tertiary over semi-consolidated Floridan limestone aquifer system at Cedar Key and Cross City is considered to be negligible, and as discussed in the previous section, SC_{nBR} is also considered to be negligible in these strata.

Subsidence due to primary compaction (SPC)

SPC is the compaction of compressible aquifer systems caused by subsurface fluid withdrawal. SPC in this paper includes the elastic and inelastic (virgin) deformation of aquitards (fine-grained deposits [clays and silts] with low permeability) and the elastic deformation of aquifers (coarse-grained deposits with moderate to high permeability) in aquifer systems. An analytical solution was developed[38,39] to simulate SPC, a coupled compaction and fluid-flow process, in a water saturated, doubly draining clay layer. The clay layer has a uniform initial pore-fluid pressure wherein only vertical water flow is permitted, and identical, instantaneous step changes in hydraulic head (or equivalent fluid pressure) occur at the bottom and top of the clay layer[38,39]. This process, based on changes in effective stress resulting from changes in pore-fluid pressure, describes the equilibration of fluid-pressure and resulting compaction, which was subsequently extended to the analysis[40] and simulation[41,42] of aquitard/confining unit drainage. This concept, known as “the aquitard drainage model”[43] has formed the theoretical basis of many successful subsidence investigations[39–41,44,45]. The time constant for the drainage and compaction of an aquitard is $\tau'_0=S'_{sk}\left(b'_0/2\right)^2/K'$, where S'_{sk}, b'_0 and K' are expressed in hydrogeologic terms denoting vertical skeletal specific storage [dimensionless], thickness [L], and vertical hydraulic conductivity [L/T] of the aquitard, respectively[40,41]. The degree of compaction in the aquitard reaches 93.1%, 99.4% and 100% of the ultimate compaction for a normalized time factor $T_v = \Delta t/\tau'_0$, where Δt is the change in time since the initial step change in hydraulic head at the upper and lower boundary of the aquitard, equals 1, 2 and ∞, respectively. SPC is deemed fully completed when T_v reaches 2 with 99.4% of the ultimate compaction realized[46]. Thus, a SPC period (Δt) can be theoretically estimated from the above expression for T_v with specified values for τ'_0 and T_v. In practice, generally SPC is dominated by inelastic deformation which occurs when the pore-fluid pressure declines result in increased effective stresses in aquitard(s) and confining unit(s) that are greater than the historical maximum effective stress (i.e., $\sigma'_o>\sigma'_c$), typically defined by the previous minimum pore-fluid pressure in those units. The deformation of the coarse-grained deposits constituting the aquifers generally proceeds only elastically for both decreases and increases in aquifer pore-fluid pressure. The elastic skeletal specific storage of the aquifers governs their deformation and is usually much smaller than S'_{sk}[47]. Elastic deformation of both aquifers and

aquitard/confining units occurs during periods of pore-fluid pressure increase (or groundwater level recovery; for example, see period II in Fig. 4) [47]. For the aquitard drainage model, inelastic compaction is governed by S'_{sk} which is typically about 1-3 magnitude orders larger than the elastic skeletal specific storage of the aquifers and the aquitards/confining units[48–51]. Notably, SPC owing to inelastic compaction of aquitards and confining units can result in appreciable land subsidence and thus, increased RSLR. In the Houston-Galveston region it was demonstrated that SPC began in about 1937 and proceeded for at least the next 63 years (Δt)[47]. SPC stopped at many borehole extensometer sites between about 2000 and 2004 (Fig. 4).

Subsidence due to creep of non-bedrock aquifer systems (SC_{nBR})

SC_{nBR} represents the deformation caused by creep behavior of sedimentary materials under a constant load. Due to the weight of the overburden (geostatic stress) and the inelastic compaction characteristics of the aquitards/confining units, about 90 percent of the deformation is permanent[52]. With regard to the degree of self-weight compression, three main sedimentation stages are defined: the clarification regime, zone-settling regime, and compression regime[53]. Quaternary, Tertiary and Cretaceous aquifer systems with a stress condition of $\sigma'_o > \sigma'_c$, remain in the compression regime and experience compaction under self-weight, which has been referred to as creep[25,54]. The path A-B in Fig. S3 shows SC_{nBR} at a constant historical maximum effective stress σ'_c due to the overburden (geostatic) stress. For an unconsolidated/semi-consolidated sediment layer with an initial thickness of H [L], SC_{nBR} can be approximated using $S_c(t)=C_\alpha H\log(t/t_0)$, which is employed in supplementary equations (S1-1–4) for the variable SC_{nBR} rate, where t_0 denotes an initial reference time for compression of creep, and t signifies time larger than or equal to t_0[25]. Taking the derivative with respect to time t of the expression above for S_c gives $\dot{S}_c = C_\alpha H/(t \ln 10)$, the subsidence rate. The decreased percentage (D_S) of $\dot{S}_c$ from t to t+Δt was derived using $[\dot{S}_c(t)-\dot{S}_c(t+\Delta t)]/\dot{S}_c(t)$ as follows[47]:

$$D_S(t)=\left(1-\frac{t}{t+\Delta t}\right)\times 100. \tag{S2}$$

For $t \gg \Delta t$, where Δt can be a short observing period such as 10–20 years, D_S approaches zero which implies that $\dot{S}_c$ is approximately a constant. In other words, the changing value of $\dot{S}_c$ over the Δt period can be ignored. For example, if a current observation period (Δt) is considered to be 10 years, 990, 1990, and 9990 years of SC_{nBR} are needed to achieve decreased percentages of 1.0, 0.5 and 0.1% of the specified subsidence rate, respectively[47]. This negligibly-variable SC_{nBR} rate[47] is used to estimate SPC.

Negligible SC_{nBR} in over semi-consolidated Tertiary and Cretaceous strata

SC_{nBR} is assumed to be negligible in Tertiary (T^{OC}) and Cretaceous (C^{OC}) strata shown in Figs. 1, 2 and Table 1. This is because the current effective stress σ'_o in T^{OC} or C^{OC} is less than its historical maximum effective stress σ'_c (Supplementary Fig. S3) owing to the outcropping or uplifting of the strata and the geological removal of the overlying Quaternary sediments Q_h and Q_p, which is referred as an overconsolidated condition[25]. Experimental investigations show that sediment creep rate with an overconsolidated stress status is significantly slower than that without[55]. In addition, the primary factor is that when compared to the age of geological strata our human observation is in such a short time period that the change in SC_{nBR} is insignificant, which has also been discussed after supplementary equation S2. For instance, the length C-D in Supplementary Fig. S3 represents the negligible subsidence of creep during a period of 100 years under effective stress σ'_o.

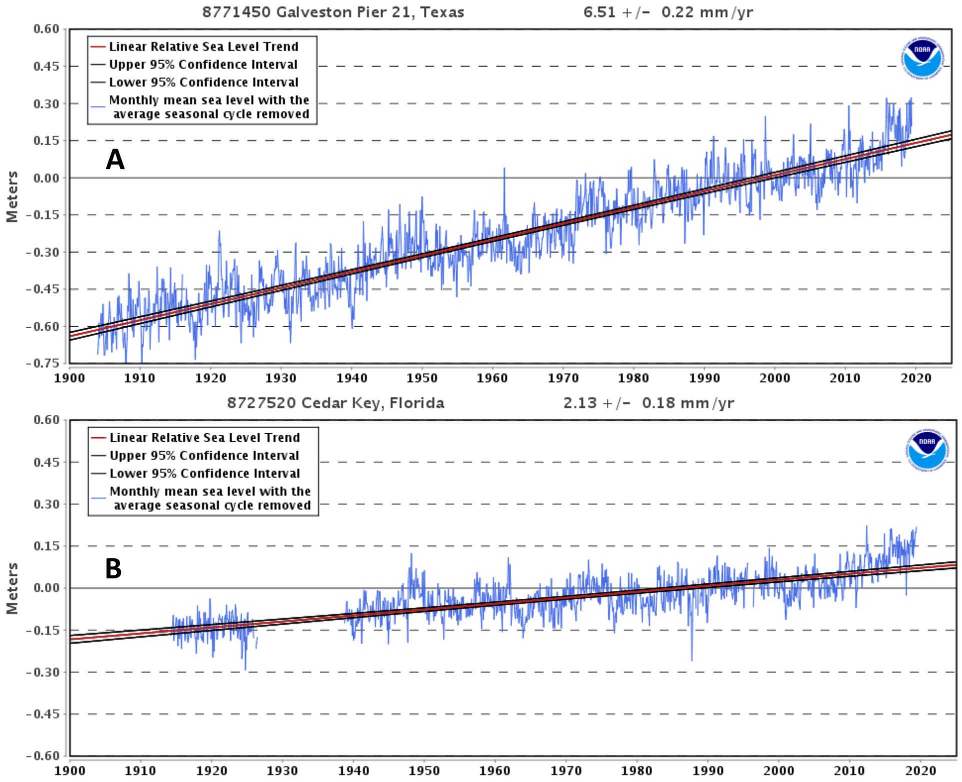

Supplementary Figure S1. Monthly mean sea level with average seasonal cycle removed, and linear trend showing relative RSLR at tide gauges: A. Galveston Pier 21; and B. Cedar Key (Image source: NOAA[35]).

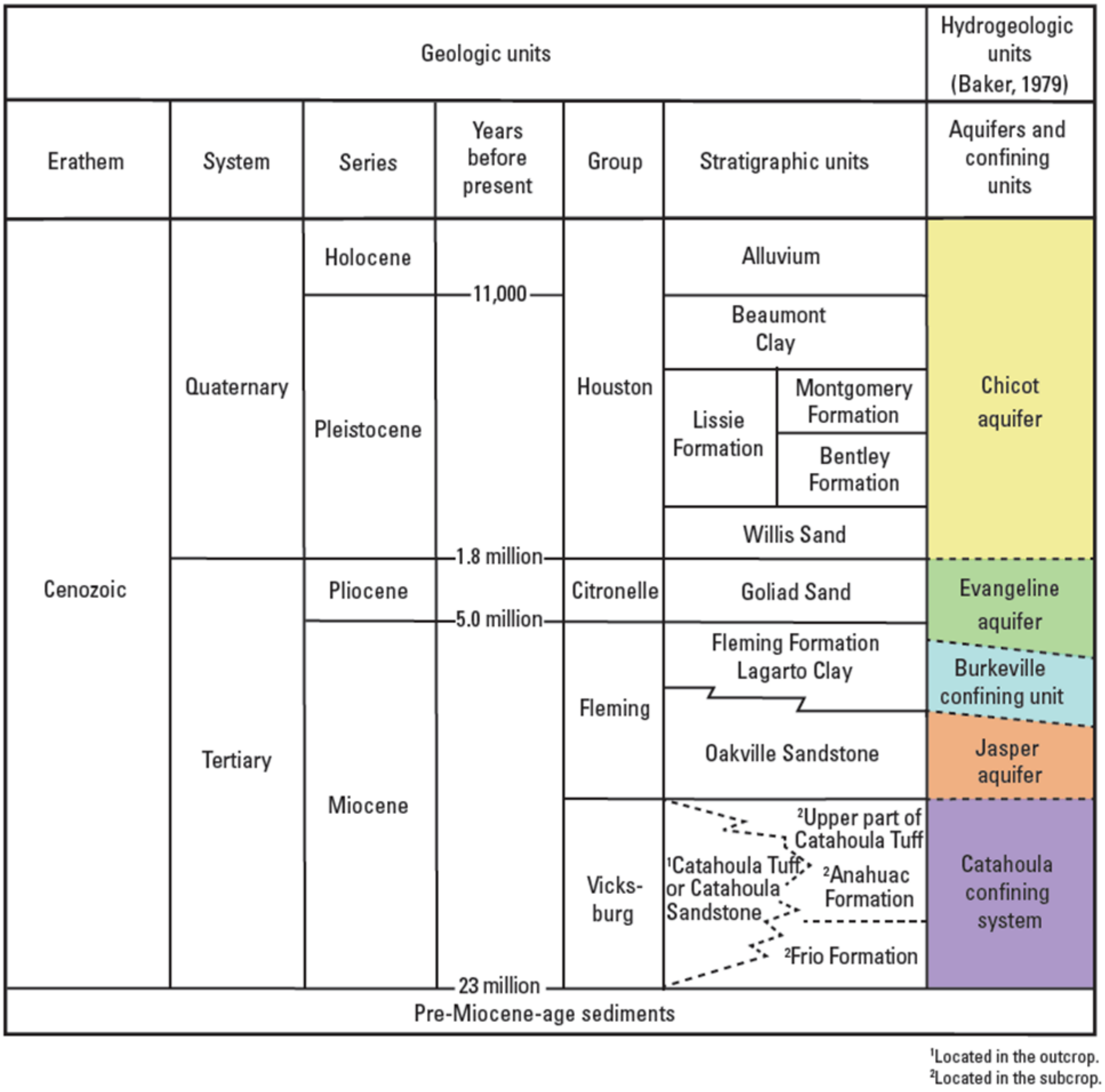

Supplementary Figure S2. Geologic and hydrogeologic units of the Gulf Coast aquifer system in the Houston-Galveston region study area, Texas[1–4]. (Map source: USGS[1])

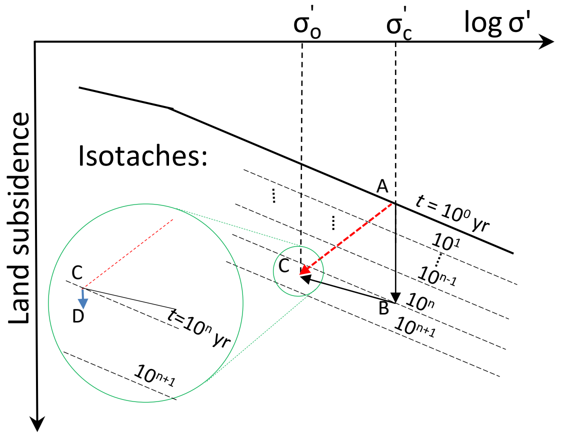

Supplementary Figure S3. Subsidence due to creep and historical changes in effective stress presented with a conceptual isotache model[25,55–57]. Path A-B shows subsidence due to 10^n year creep under a given overburden pressure σ'_c. Path B-C displays rebound due to an instant overburden removal at the age of 10^n years and reduction of effective stress to σ'_o from σ'_c. Path A-C represents combination of both the rebound and creep processes that may occur simultaneously. Path C-D at σ'_o shows insignificant subsidence of creep during 100 years (e.g., 1900 – 2000) from 10^n years to 10^n + 100 years.

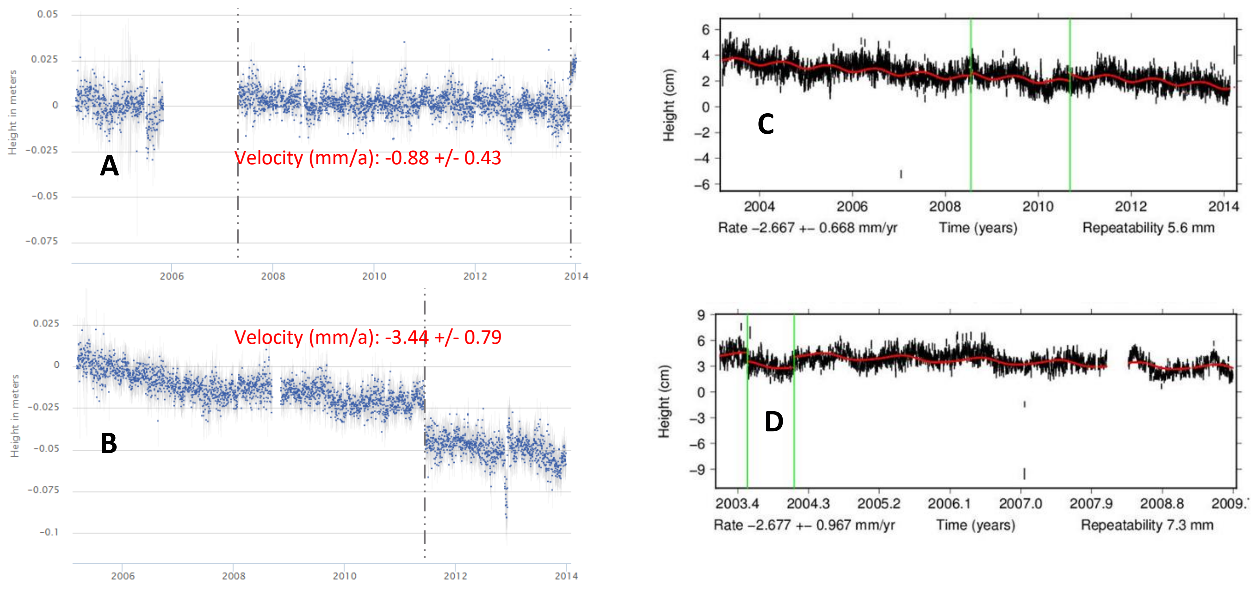

Supplementary Figure S4. Land subsidence (LS) derived from GPS stations: A. Subsidence of bedrock systems (LS_{BR}) of 0.88 mm/a from GPS station XCTY at Cross City, Florida[34]; B. Total LS of 3.44 mm/a, which is the sum of LS_{BR} and SC_{nBR}, from GPS station TXGA, Texas (see location in Supplementary Fig. S6)[34]; C. LS_{BR} of 2.67 mm/a from GPS station SG32 at College Station, Texas[33]; D. LS_{BR} of 2.68 mm/a from GPS station LDBT near Lake Bastrop, Texas. (Observations in B and D: Black points with error bars; Fit: Red points; and breaks (or discontinuities) in position: Green bars[33]). (Image source: SONEL[34] for A & B and Courtesy NASA/JPL-Caltech[58] for C & D)

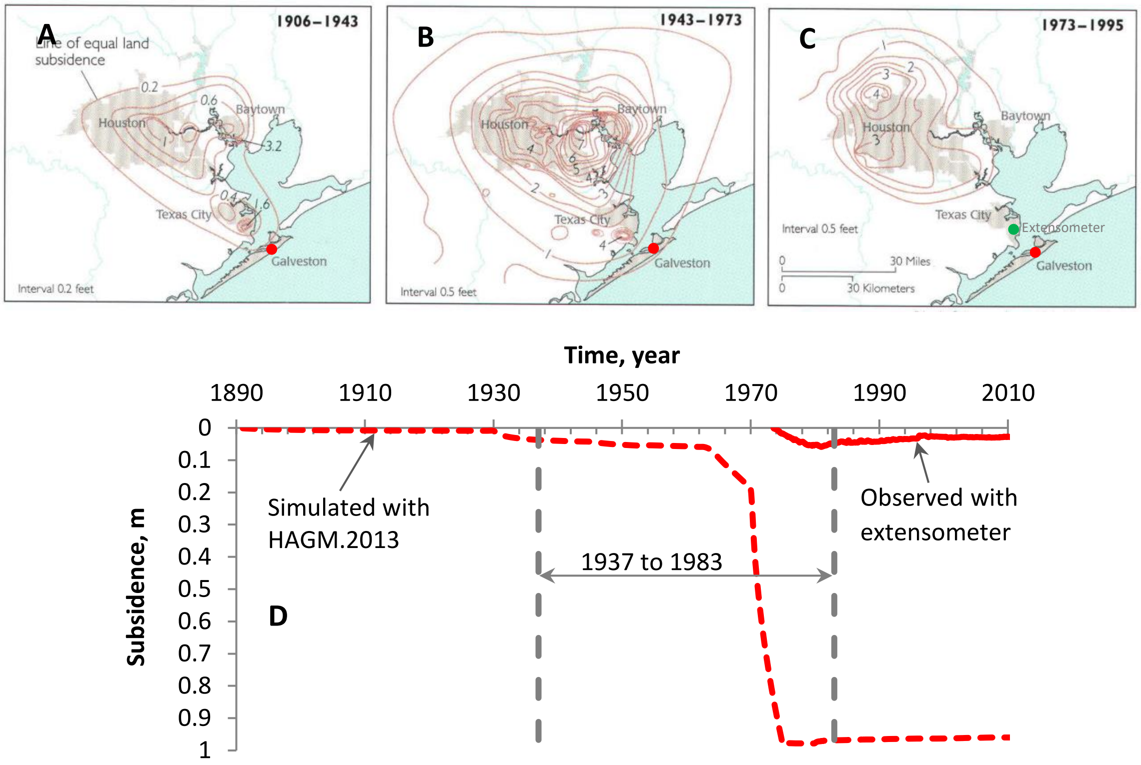

Supplementary Figure S5. Subsidence due to primary compaction (SPC) measured and simulated in groundwater withdrawal areas close to Galveston Pier 21 (modified from[45]: A. B. and C . measured subsidence contours; D. Simulated subsidence at Texas City, Galveston County, Texas using HAGM.2013[5] and observed subsidence at the Texas City–Moses Lake borehole extensometer (see Supplementary Fig. S6 for location). (Image source for A, B & C: USGS[45])

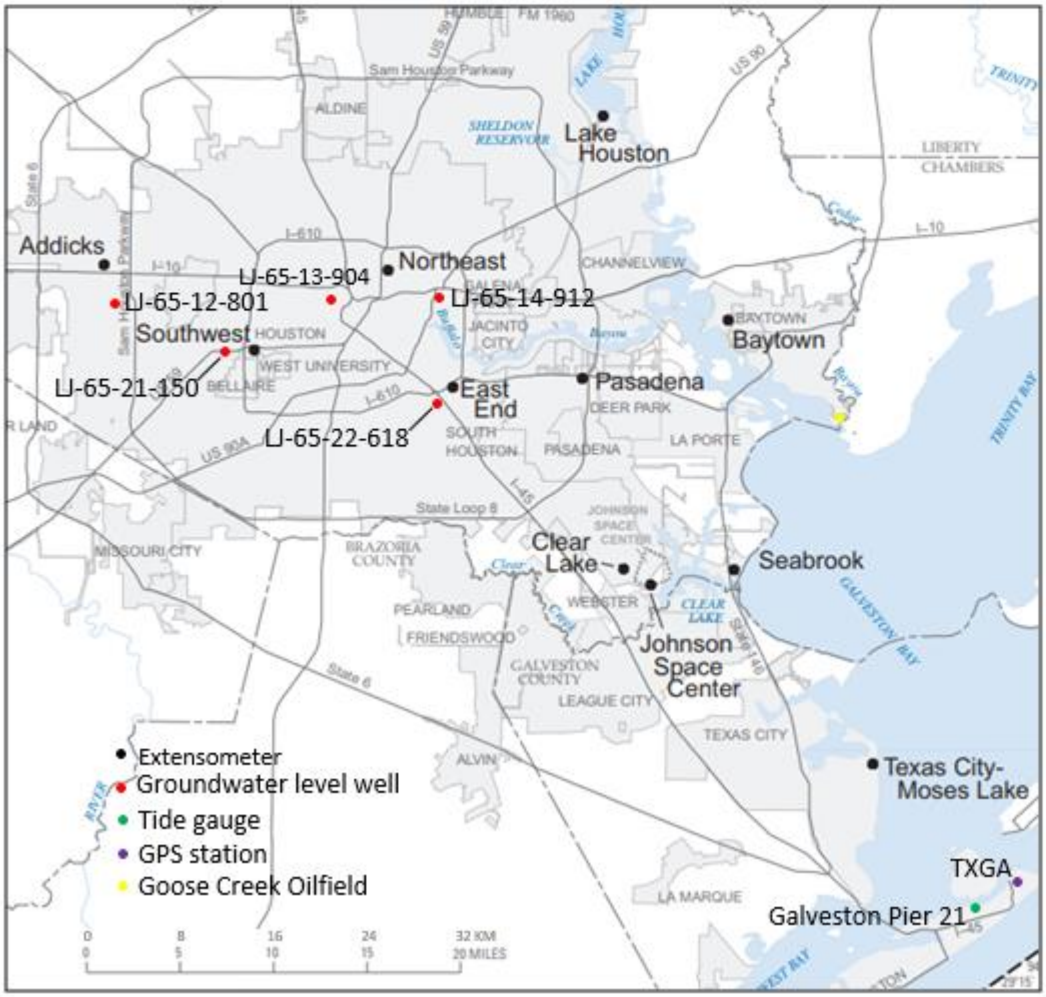

Supplementary Figure S6. Location of borehole extensometer sites and selected groundwater level monitoring well sites, Houston Galveston region, Texas (modified from[59]). (Note: The location of wells LJ-65-21-229 and LJ-65-21-227 is at the same location of extensometer Southwest) (Base map soure: USGS[59])

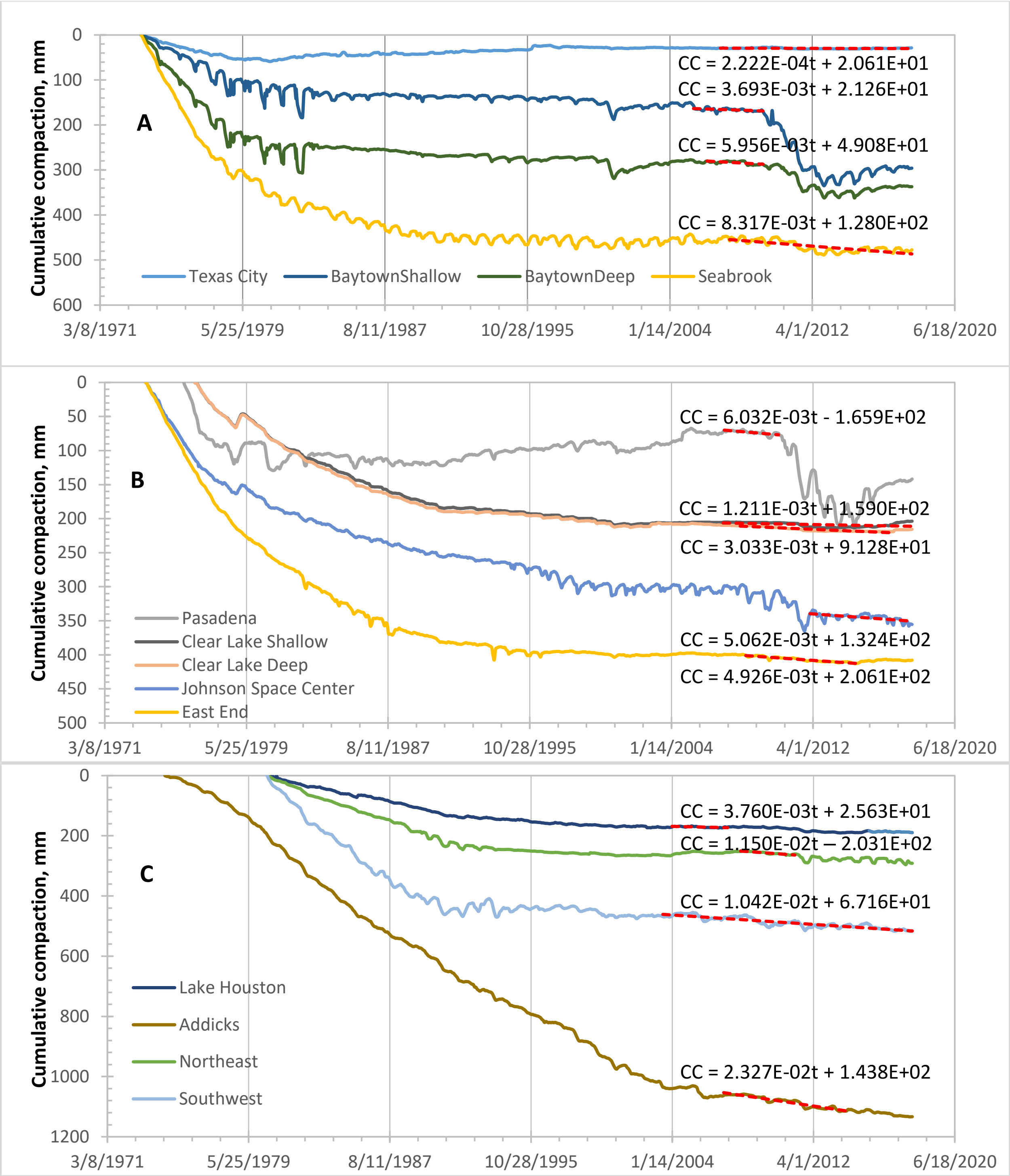

Supplementary Figure S7. Time series of observed cumulative compaction (CC; Data source: USGS) and linear trendlines (dashed red lines) of CC for SCnBR (or creep) at 13 borehole extensometer sites in the Houston-Galveston region located: A. Very near or on the coast; B. Near the coast and generally near bayous; and C. Inland, farther from the coast. The compaction period for SCnBR for each site is given in Supplementary Table S1. The slopes in the trendline equations are in mm/d. (modified from[47])

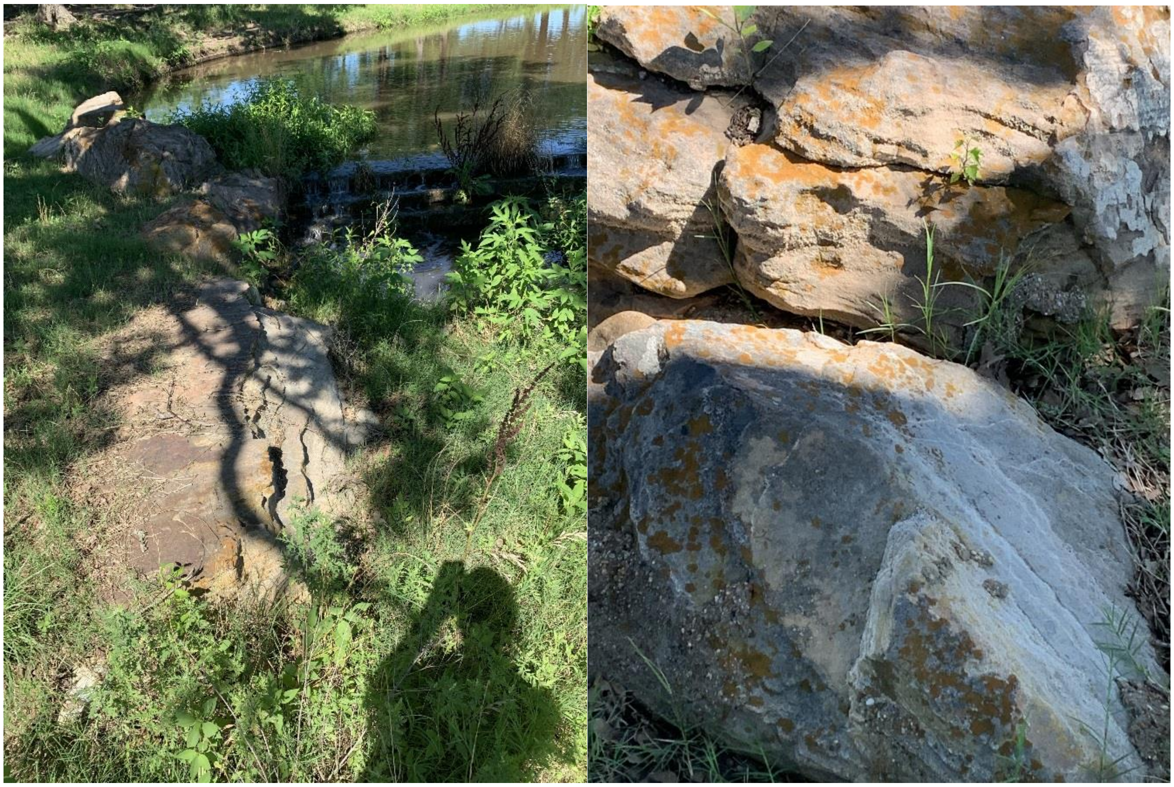

Supplementary Figure S8. Tertiary silty sandstone outcrops (Yegua Formation) at College Station, Texas.

Extensometer		Well #	Aquifer	SC_{nBR} appearance period		Groundwater level Trend		SC_{nBR} rate	
	**Depth, m**			starting date	ending date	m/d	m/a	mm/d	mm/a
Texas City	244	KH-64-33-901	Chicot	1/24/2008	1/14/2017	-8.73E-05	-0.03	2.222E-04	0.08
Seabrook	421	LJ-65-32-519	Chicot	1/25/2008	1/14/2017	-2.77E-05	-0.01	8.317E-03	3.04
		LJ-65-32-630	Evangeline			-7.67E-05	-0.03		
Space Center Clear Lake	239	LJ-65-42-422	Chicot	1/25/2007	12/20/2017	9.09E-05	0.03	5.062E-03	1.85
	937[*]	LJ-65-42-424	Evangeline			6.79E-05	0.02	3.033E-03	1.11
Baytown Shallow	131	LJ-65-16-933	Chicot	5/26/2005	5/28/2009	3.82E-04	0.14	3.630E-03	1.33
Baytown Deep	450	LJ-65-16-931	Evangeline	1/11/2007		9.21E-05	0.03	5.956E-03	2.17
Addicks	549	LJ-65-12-729	Chicot	10/1/2007	5/15/2014	2.73E-04	0.10	2.327E-02	8.49
		LJ-65-12-726	Evangeline			0.00E+00	0.00		
EastEnd	304	LJ-65-22-623	Chicot	7/27/2007	1/13/2015	-2.72E-04	-0.10	4.926E-03	1.80
		LJ-65-22-622	Evangeline			5.64E-04	0.21[**]		
Northeast	663	LJ-65-14-745	Chicot	1/4/2008	3/1/2011	5.27E-04	0.19	1.150E-02	4.20
		LJ-65-14-746	Evangeline			1.07E-03	0.39[***]		
Pasadena	864	LJ-65-23-321	Chicot	1/5/2007	1/5/2011	3.76E-06	0.00	6.032E-03	2.20
		LJ-65-23-326	Evangeline	2/6/2007	3/30/2010	8.39E-04	0.31[****]		
Lake Houston	591	LJ-65-07-902	Chicot	1/7/2004	4/4/2007	8.38E-05	0.03	3.760E-03	1.37
		LJ-65-07-908	Evangeline			1.24E-04	0.05		
Southwest	719	LJ-65-21-229	Chicot	9/18/2003	10/18/2018	-1.13E-04	-0.04	1.042E-02	3.80
		LJ-65-21-227	Evangeline			4.59E-04	0.17		

[*]: Clear Lake Deep is 937 m deep and Clear Lake Shallow is 530 m deep.
[**]: The difference of groundwater level between -42.75 m on 5/7/2008 and -42.77 m on 11/3/2014 is 0.02 m.
[***]: The difference of groundwater level between -54.14 m on 1/4/2008 and -54.18 m on 3/1/2011 is 0.04 m.
[****]: The difference of groundwater level between -40.03 m on 2/6/2007 and -40.17 m on 3/30/2010 is 0.14 m.

Supplementary Table S1. SC_{nBR} (creep) appearance periods based on groundwater level trend at or near extensometer sites in the Houston-Galveston region (modified from[47])

Tide gauge	Galveston Pier 21		Cedar Key	
	Linear trend of RSLR from NOAA	LS_{BR} + SC_{nBR} from GPS TXGA	Linear trend of RSLR from NOAA	LS (LS_{BR} + SC_{nBR}) from GPS XCTY
Constant (linear) rate, mm/a	6.51	3.44	2.13	0.88
ASLR, mm/a	3.07		1.25	
Difference, mm/a	1.82*			
*: 3.07 mm/a has 146% difference relative to 1.25 mm/a.				

Supplementary Table S2. ASLR values obtained with linear RSLR trend method

Supplementary References

1. Kasmarek, M. C., Ramage, J. K. & Johnson, M. R. *Water-level altitudes 2016 and water-level changes in the Chicot, Evangeline, and Jasper aquifers and compaction 1973–2015 in the Chicot and Evangeline aquifers, Houston–Galveston region, Texas*. (USGS, 2015). doi:10.3133/SIM3308
2. Sellards, E. H., Adkins, W. S. & Plummer, F. B. The Geology of Texas: Volume 1 Stratigraphy. *Univ. Texas Bull.* 1007 (1932).
3. Baker, E. T. *Stratagraphic and Hydrogeologic framework of Part of the Texas Coastal Plain*. (Texas Department of Water Resources, 1979).
4. Meyer, W. R. & Carr, J. E. A digital model for simulation of ground-water hydrology in the Houston area, Texas. *Texas Dapartment Water Resour.* 76 (1979).
5. Kasmarek, M. C. *Hydrogeology and Simulation of Groundwater Flow and Land-Surface Subsidence in the Northern Part of the Gulf Coast Aquifer System, Texas, 1891–2009*. (U.S. Geological Survey, Scientific Investigation Report 2012-5154, 2013).
6. Coplin, L. S. & Galloway, D. L. Houston-Galveston, Texas: managing coastal subsidence. in *Land subsidence in the United States* 35–46 (US Geological Survey Circular, 1182, 1999).
7. Scott, T. M. *Text To Accompany the Geology Map of Florida*. (2001).
8. Dall, W. H. & Harris, G. D. *Correlation papers - Neocence*. (1892).
9. Puris, H. S. *Contribution to the study of the Miocene and the Florida panhandle*. (1953).
10. Puris, H. S. *Strategraphy and zonation of the Ocala group*. (1957).
11. Miller, J. A. *Hydrogeologic framework of the Floridan aquifer system in Florida and parts of Georgia, Alabama and South Carolina*. (1986).
12. Eargle, S. H. *Nomenclature of formations of Claiborne Group, Middle Eocene, Coast plain of Texas*. (1968).
13. George, P. G., Mace, R. E. & Petrossian, R. Aquifers of Texas. *Texas Water Dev. Board* **380**, 1–182 (2011).
14. Bureau of Economic Geology. *Geologica map of Texas (1:500,000)*. (1992).
15. Parris, A. *et al. Global Sea Level Rise Scenarios for the United States National Climate Assessment*. (2012).
16. USACE. *INCORPORATING SEA LEVEL CHANGE IN CIVIL WORKS PROGRAMS*. (2019).
17. Ezer, T. & Corlett, W. B. Is sea level rise accelerating in the Chesapeake Bay ? A demonstration of a novel new approach for analyzing sea level data. **39**, 1–6 (2012). doi: 10.1029/2012GL053435
18. USACE. Galveston's Bulwark Against the Sea: History of the Galveston Seawall. (1981).
19. Yip, T. L., Zhang, D.-H. & Chwang, A. T. Environmental and Safety Considerations for Design of a Perforated Seawall. *Proc. Int. Offshore Polar Eng. Conf.* **12**, 758–763 (2002).
20. Folger, P. & Carter, N. T. *Sea - Level Rise and U . S . Coasts : Science and Policy Considerations*. (2016).
21. National Committee on Levee Safety. *RECOMMENDATIONS FOR A NATIONAL LEVEE SAFETY PROGRAM, A Report to Congress from the National Committee on Levee Safety (DRAFT)*. (2009).
22. Harder, L. F., Basham, D. L., Verigin, S. W. & Williams, W. D. ENGINEERING PERSPECTIVES FOR A NATIONAL LEVEE SAFETY PROGRAM Abstract : What

We Have Learned About the Nation ' s Levees. 1–14 (2009).
23. IPCC. *Climate Change 2014: Synthesis Report. Contribution of Working Groups I, II and III to the Fifth Assessment Report of the Intergovernmental Panel on Climate Change*. (2014). doi:10.1017/CBO9781107415324
24. FEMA. Recommended Residential Construction for Coastal Areas: Building on Strong and Safe Foundations. **242**, (2009).
25. Taylor, D. W. *Research on consolidation of clays, Department of Engineering, Massachusetts Institute of Technology, Cambridge, Mass.* **Serial 82**, 147 (1942).
26. Doherty, J. *PEST, Model-independent parameter estimation-User manual (5th ed.).* (2004).
27. Moser, S. C. *et al.* Coastal Zone Development and Ecosystems. in *Climate Change Impacts in the United States: The Third National Climate Assessment* (ed. J. M. Melillo, Terese (T.C.) Richmond, and G. W. Y.) 579–618 (2014). doi:doi:10.7930/J0MS3QNW. http://nca2014.globalchange.gov/report/regions/coasts
28. Benford, B., Demets, C. & Calais, E. GPS estimates of microplate motions, northern Caribbean: Evidence for a Hispaniola microplate and implications for earthquake hazard. *Geophys. J. Int.* **191**, 481–490 (2012). doi:10.1111/j.1365-246X.2012.05662.x
29. Goudarzi, M. A., Cocard, M. & Santerre, R. Present-Day 3D Velocity Field of Eastern North America Based on Continuous GPS Observations. *Pure Appl. Geophys.* **173**, 2387–2412 (2016). doi:10.1007/s00024-016-1270-7
30. Mitrovica, J. X., Milne, G. A. & Davis, J. L. Glacial isostatic adjustment on a rotating earth. *Geophys. J. Int.* **147**, 562–578 (2001).
31. Chigira, M. Long-term gravitational deformation of rocks by mass rock creep. *Eng. Geol.* **32**, 157–184 (1992).
32. Boukharov, G. N., Chanda, M. W. & Boukharov, N. G. The three processes of brittle crystalline rock creep. *Int. J. Rock Mech. Min. Sci.* **32**, 325–335 (1995). doi:10.1016/0148-9062(94)00048-8
33. JPL. GNSS Time Series. Available at: https://sideshow.jpl.nasa.gov/post/series.html. (Accessed: 21st June 2019)
34. SONEL. SONEL. Available at: https://www.sonel.org/?lang=en. (Accessed: 21st June 2019)
35. NOAA. Sea Level Trends. Available at: https://tidesandcurrents.noaa.gov/sltrends/sltrends.html. (Accessed: 21st June 2019)
36. Bush, P. W. & Johnston, R. H. *Ground-water hydraulics, regional flow, and ground-water development of the Floridan aquifer system in Florida and in parts of Georgia, South Carolina, and Alabama*. *US Geological Survey Profesional Paper* (1988). doi:10.3133/pp1403C
37. Bellino, J. C., Kuniansky, E. L., O'Reilly, A. M. & Dixon, J. F. *Hydrogeologic setting, conceptual groundwater flow system, and hydrologic conditions 1995–2010 in Florida and parts of Georgia, Alabama, and South Carolina*. *US Geological Survey Scientific Investigations Report 2018-5030* (2018). doi:10.3133/sir20185030
38. Terzaghi, K. Settlement and consolidation of clay. in *Principles of Soil Mechanics, vol. IV* 874–878 (McGraw-Hill, 1925).
39. Terzaghi, K. Principles of soil mechanics, IV-Settlement and consolidation of clay. *Eng. News-Record* **95**, 974–978 (1925).
40. Riley, F. S. Analysis of borehole extenso meter data from central California. in

International Association of Scientific Hydrology Publication 89 423–431 (1969).
41. Helm, D. C. One-dimensional simulation of aquifer system compaction near Pixley, Calif., part 1. Constant parameters. *Water Resour. Res.* **11**, 465–478 (1975).
42. Helm, D. C. One-dimensional simulation of aquifer system com- paction near Pixley, California: 2. Stress-dependent parameters. *Water Resour. Res.* **12**, 375–391 (1976).
43. Helm, D. C. Field-based computational techniques for predicting subsidence due to fluid withdrawal.pdf. *Geol. Soc. Am. Rev. Eng. Geol.* **VI**, 1–22 (1984).
44. Poland, J. F. Land subsidence in the San Joaquin Valley and its effect on estimates of ground-water resoruces. in *lASH Publication 52* 325–335 (International Association of Scientific Hydrology, 1960).
45. Galloway, D. L., Jones, D. R. & Ingebritsen, S. E. *Land subsidence in the United States*. (U.S. Geological Survey, Circular 1182, 1999).
46. Liu, Y. & Li, J. MODFLOW No-Delay Flow Simulation for Low Permeable Confining Units. in *the Proceedings of World Environmental & Water Resources Congress 2016, West Palm Beach, Florida, MAY 22-26, 2016* (2016). doi:10.1061/9780784479865.040
47. Liu, Y., Li, J. & Fang, Z. N. Groundwater Level Change Management on Control of Land Subsidence Supported by Borehole Extensometer Compaction Measurements in the Houston-Galveston Region, Texas. *Geosciences* **9**, 19 (2019).doi: 10.3390/geosciences9050223
48. Pope, J. P. & Burbey, T. J. Multiple-aquifer characterization from single borehole extensometer records. *Ground Water* **42**, 45–58 (2004).
49. Liu, Y. & Helm, D. C. Inverse procedure for calibrating parameters that control land subsidence caused by subsurface fluid withdrawal: 2. Field application. *Water Resour. Res.* **44**, (2008).doi:10.1029/2007WR006606
50. Sneed, M. & Galloway, D. L. *Aquifer-system compaction and land subsidence*. (U.S. Geol. Surv. Water Resour. Invest. Rep., 00– 4015, 2000).
51. Riley, F. S. Mechanics of aquifer systems-The scientific legacy of Joseph F. Poland. in *Land subsidence case studies and current research: Proceedings of the Dr. Joseph F. Poland symposium on land subsidence* 13–27 (Association of Engineering Geologists Special Publication No. 8, 1998).
52. Gabrysch, R. K. & Bonnett, C. W. *Land-surface subsidence in the Houston-Galveston Region, Texas*. (1975).
53. Fitch, B. Kynch theory and compression zones. *AIChE J.* **29**, 940–942 (1983).
54. Taylor, D. W. & Merchant, W. A. A theory of clay consolidation accounting for secondary compression. *J. Math. Phys.* **19**, 167–185 (1940).
55. Yuan, Y., Whittle, A. J. & Nash, D. F. T. Model for Predicting and Controlling Creep Settlements with Surcharge Loading. *Deform. Charact. Geomaterials* 931–938 (2015). doi:10.3233/978-1-61499-601-9-931
56. Bjerrum, L. Engineering geology of Norwegian normally consolidated marine clays as related to settlements of buildings. *Géotechn.* **17**, 81–118 (1967).
57. Kooi, H., Bakr, M., Lange, G. D., Haan, E. D. & Erkens, G. *User Guide to DUB-CR - A MODFLOW package for land subsidence and aquifer system compaction that includes creep*. *Regulation* (2018).
58. Heflin, M. *et al.* GPS Time Series. (2018). https://sideshow.jpl.nasa.gov/post/series.html
59. Kasmarek, M. K. & Lanning-Rush. Water-level altitudes 2003 and water-level changes in the Chicot , Evangeline, and Jasper aquifers and compaction 1973 – 2002 in the Chicot

and Evangeline aquifers, Houston-Galveston region, Texas. *USGS Open-file Rep. 03-109* (2003).
